# Modelling eCPR eligibility after out-of-hospital cardiac arrest: a 10-year retrospective temporospatial analysis from a mixed urban-rural region in Germany

**DOI:** 10.1186/s13049-026-01681-7

**Published:** 2026-09-09

**Authors:** Clemens Grimm, Hanna Illian, Nils Kunze-Szikszay, Hanna Haus, Claudius Balzer, Markus Roessler

**Affiliations:** 1 Department of Anaesthesiology, University Medical Centre Göttingen, Robert-Koch-Str. 40, Göttingen, 37073 Germany; 2 Department of Anaesthesiology, Operative Intensive Care Medicine and Pain Therapy, Christliches Klinikum Paderborn, Husener Str. 46, Paderborn, 33098 Germany; 3 Bielefeld University, Medical School and University Medical Centre OWL, Klinikum Bielefeld - Mitte, Centre of Anaesthesiology, Intensive Care, Emergency Medicine and Pain Therapy, Teutoburger Str. 50, Bielefeld, 33604 Germany

**Keywords:** Extracorporeal membrane oxygenation, Out-of-hospital cardiac arrest, Geospatial modelling, Extracorporeal life support, Extracorporeal cardiopulmonary resuscitation

## Abstract

**Background:**

Survival with good neurological function after out-of-hospital cardiac arrest (OHCA) remains low. Extracorporeal cardiopulmonary resuscitation (eCPR) may benefit selected patients, but it is unclear how many patients in a mixed urban-rural European region would be eligible, whether regional case volumes justify dedicated eCPR programmes, and whether an in-hospital strategy after intra-arrest transport can meet a 60-minute low-flow target.

**Methods:**

We conducted a retrospective cohort study of OHCA cases in the city and county of Göttingen, Germany (January 2015–February 2025). eCPR eligibility was assessed using a local protocol and a stricter modified ELSO definition. Eligibility was first determined at emergency medical services (EMS) arrival and then re-evaluated for three hypothetical intra-arrest transport strategies in which transport was initiated after 10, 15, or 20 minutes of on-scene conventional cardiopulmonary resuscitation (cCPR). Scene-to-hospital travel times were modelled and patients were classified according to whether they would be eligible for eCPR upon hospital arrival. A complementary analysis estimated time from collapse to hypothetical eCPR initiation under an on-scene scenario, split into simultaneous dispatch of a dedicated eCPR team (S1a) and delayed dispatch after 10 minutes of on-scene evaluation by the primary EMS team (S1b), and an in-hospital scenario following intra-arrest transport (S2), incorporating documented timing of sustained return of spontaneous circulation (ROSC).

**Results:**

Of 2,218 OHCA cases, 1,696 registry-documented patients were analysed (median age 71.5 years; 67.5% male; shockable rhythm 20.5%). At EMS arrival, 348 patients (20.5%) met local and 149 (8.8%) modified ELSO criteria. With transport initiated after 10/15/20 minutes of on-scene cCPR, 11.1%/9.8%/8.8% (local) and 3.8%/3.1%/2.8% (modified ELSO) would have been eligible at hospital arrival. Extrapolated regional case volume was approximately 46 (local) and 20 (modified ELSO) eligible patients per year at EMS arrival. In patients who met local eCPR criteria the number of patients with sustained ROSC exceeded those without at 13.4 minutes after EMS arrival. Median estimated time from collapse to eCPR was 37 minutes under S1a (94.8% within 60 minutes) and 54 minutes under S1b (71.6% within 60 minutes) versus 75 minutes under S2 (<1% within 60 minutes). After excluding patients with ROSC before hypothetical eCPR initiation, 47.4% (S1a) and 31.6% (S1b) in the local cohort and 36.9% (S1a) and 24.8% (S1b) in the modified ELSO cohort would both still have required and could have received on-scene eCPR within 60 minutes.

**Conclusions:**

A substantial number of OHCA patients in our region met eCPR eligibility criteria, but hospital arrival did not equate to eCPR initiation. Given realistic procedural time intervals, in-hospital eCPR following intra-arrest transport rarely met the 60-minute target, whereas on-scene eCPR might have remained feasible for most eligible patients.

**Supplementary Information:**

The online version contains supplementary material available at https://doi.org/10.1186/s13049-026-01681-7.

## Background

Extracorporeal cardiopulmonary resuscitation (eCPR) is an advanced resuscitative strategy that utilises veno-arterial extracorporeal membrane oxygenation (VA-ECMO) to provide circulatory support during cardiac arrest. In contrast to conventional cardiopulmonary resuscitation (cCPR), eCPR delivers oxygenated blood to vital organs, potentially extending the window for definitive treatment of the underlying cause of arrest [1].

During prolonged resuscitative efforts cCPR is often inadequate, especially regarding cerebral oxygen supply [2]. In contrast, eCPR may significantly improve survival and neurological outcomes in patients who fail to achieve return of spontaneous circulation (ROSC) with standard interventions in a timely manner [3]. However, despite promising results from randomised trials and observational data, the widespread implementation of eCPR remains limited, hindered by logistical, financial, and ethical challenges. Recent meta-analyses indicated survival benefits for eCPR in both in-hospital and out-of-hospital cardiac arrest. However, uncertainty regarding its definitive benefit in terms of patient-oriented outcomes is still high [4].

The optimal setting for the implementation of eCPR remains a subject of ongoing debate [5, 6]. In-hospital initiation of eCPR for OHCA cases has been adopted in some centres, but outcomes have varied considerably [7]. Consequently, there is growing interest in initiating eCPR earlier (e.g. during the prehospital phase of care) as poor outcomes after OHCA are closely linked to prolonged low-flow intervals [8]. In recent years, the feasibility and potential benefits of prehospital eCPR provided by emergency medical services (EMS) have been the focus of investigations [9]. Notably, the SUB30, CHEER3 and PRECARE studies demonstrated both the practicability and benefits of this approach [9–11]. Currently, a large-scale, multicentre trial is underway in the Netherlands to further evaluate prehospital eCPR [12].

However, inclusion criteria for eCPR remain variable across studies, and accurately identifying patients who are most likely to benefit from this resource-intensive therapy continues to pose a significant challenge [13–16]. Retrospective datasets offer valuable opportunities to explore this question, allowing for the identification of potentially eligible patients who may have benefited from eCPR had it been available—especially when considering the operational and economic constraints of real-world systems [17].

Inspired by the predictive modelling framework proposed by Vos et al., this study aims to retrospectively evaluate how many patients in a large existing dataset might have met various clinical and temporal eligibility criteria for eCPR [18]. We aimed to provide objective information for EMS system planning and support strategic decisions regarding the allocation of limited resources for future eCPR programmes.

## Methods

### Study design and setting

This retrospective cohort study analysed all OHCA data within the city and administrative district of Göttingen that were documented in the regional database for (H)EMS missions as well as in the German Resuscitation Registry (*Deutsches Reanimationsregister*) collected from January 1st 2015 to February 2nd 2025. Detailed characteristics regarding the study setting can be found in Supplementary Table S5.

The study was approved by the Ethics Committee of the University Medical Centre Göttingen (reference number 10/7/25). Individual informed consent was neither required nor feasible. The reporting of this study followed the STROBE guideline.

The analysis examined eCPR eligibility at two distinct time points: (1) on-scene, based on patient characteristics and initial resuscitation data, and (2) upon hospital arrival, as modelled under three hypothetical EMS transport strategies. The aim of the study was to assess the potential for eCPR at various time points during OHCA treatment by EMS.

### Inclusion criteria

The German Resuscitation Registry captures all EMS-confirmed out-of-hospital cardiac arrests in participating regions, irrespective of whether resuscitation was initiated. For the present analysis, only patients in whom resuscitation was attempted or continued by EMS were included, as only these represent potential eCPR candidates.

Under the local institutional protocol for in-hospital eCPR, based on the German national consensus paper [19], patients were considered eligible if they were aged 16 to 75 years, had a witnessed collapse, a presumed non-traumatic cause of arrest, and a no-flow time of no more than 10 minutes.

A second, stricter eligibility definition was based on a modified version of the 2021 ELSO (Extracorporeal Life Support Organization) Interim Guideline Consensus Statement adapted to the variables available in our dataset [20]. Following this definition, patients were considered eligible if they were aged 16 to 69 years, had a witnessed collapse, a presumed non-traumatic cause of arrest, a no-flow time of 5 minutes or less, and an initial shockable rhythm (ventricular fibrillation or pulseless ventricular tachycardia) or pulseless electrical activity.

**Fig. 1:**
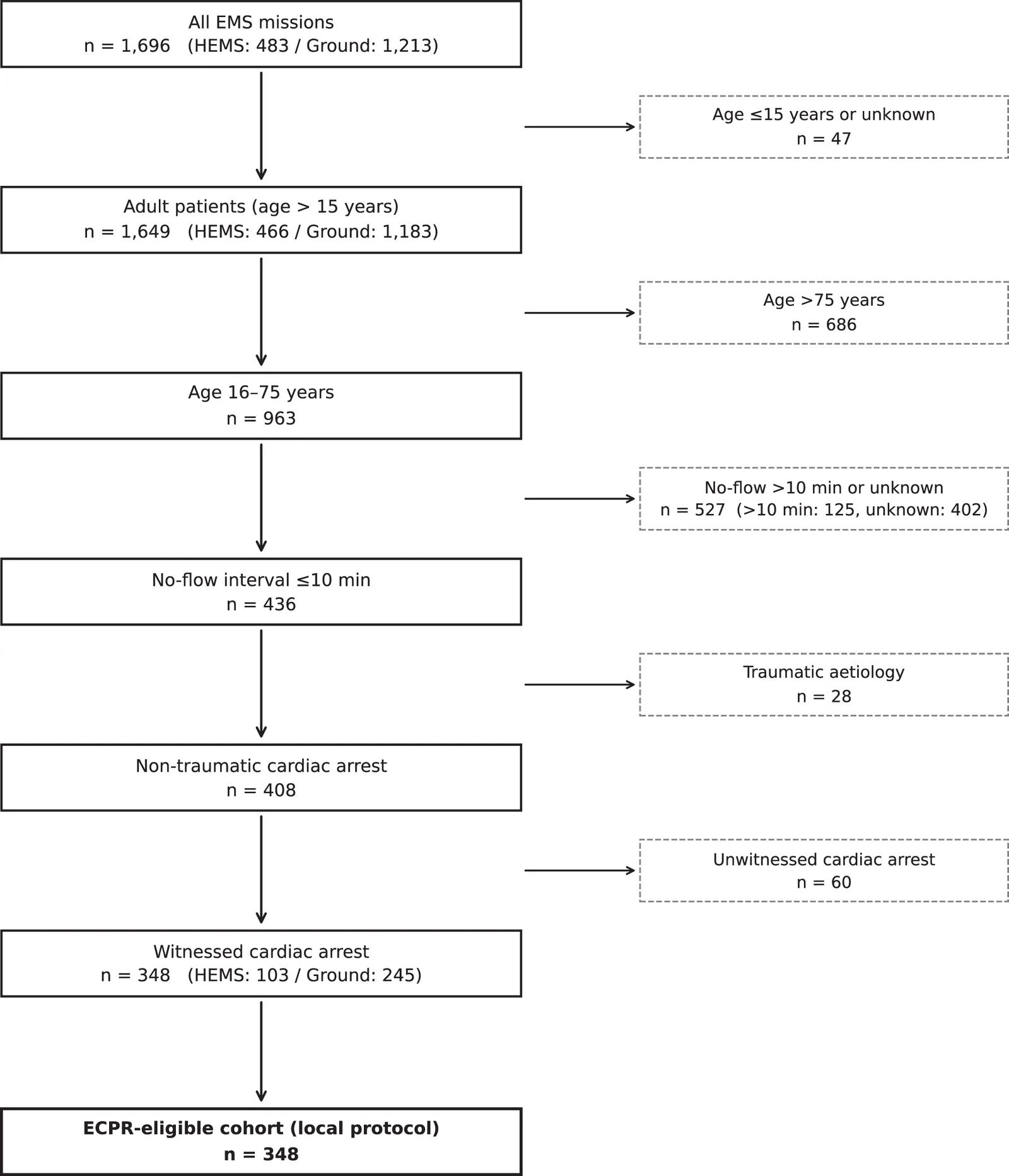
Patient selection—local eCPR protocol

**Fig. 2:**
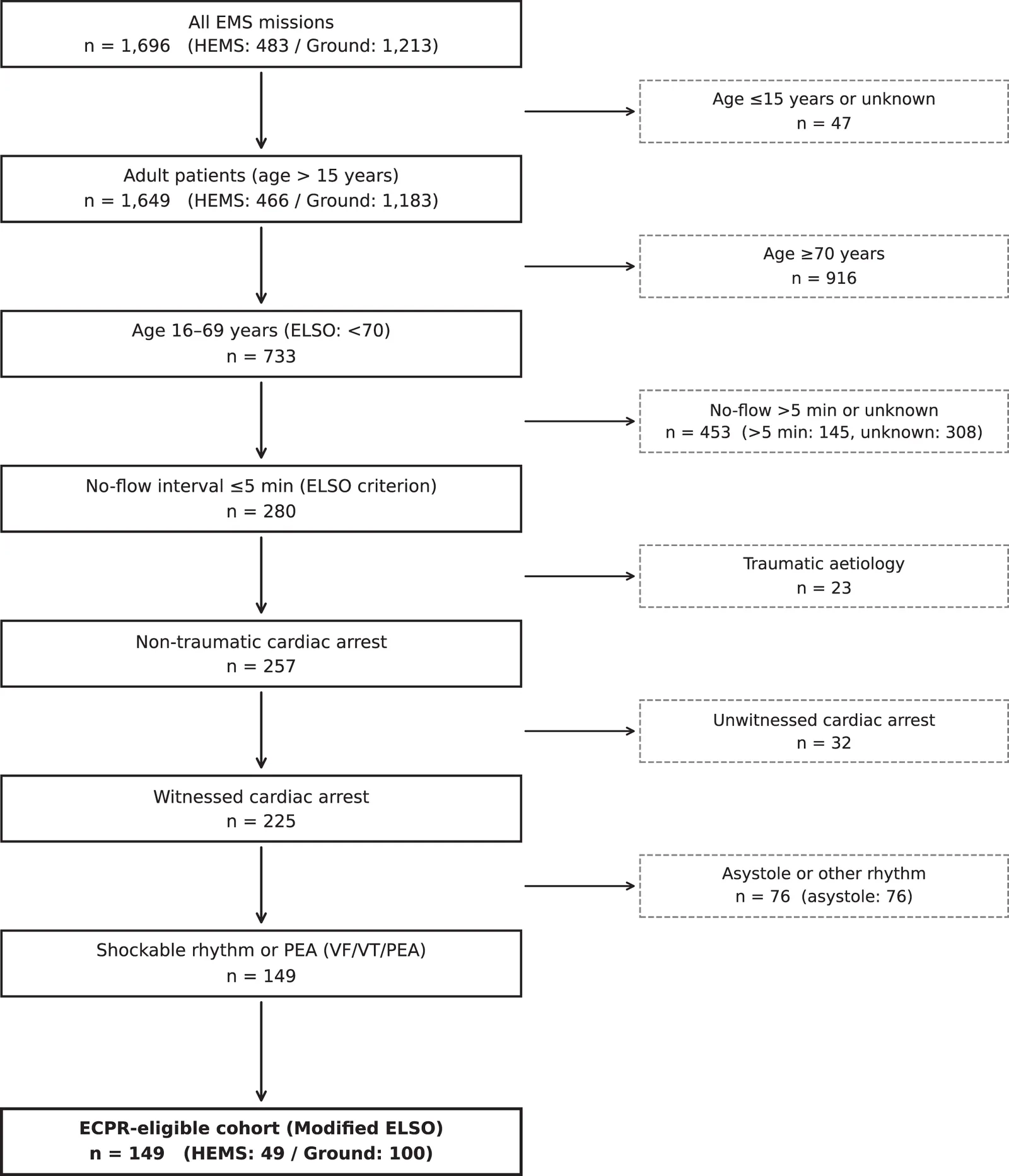
Patient selection—modified ELSO cohort

Study flowcharts of both cohorts are provided in Figs. 1 and 2, respectively.

### Study size

No sample-size calculation was performed. The study size was determined by the number of OHCA cases documented in the regional EMS database and German Resuscitation Registry during the predefined study period.

### Definitions

The time from collapse or, if unavailable, from the earliest recorded dispatch time to the arrival of the first EMS team was defined as the EMS response time. For patients who arrested in the presence of EMS personnel, the response time was set to zero as professional resuscitation could be initiated immediately. Treatment time refers to the period of on-scene resuscitation before a hypothetical decision to initiate transport, modelled at 10, 15, or 20 minutes depending on the scenario. Transport time represented the interval from departure from the scene to arrival at the regional eCPR-capable hospital (University Medical Centre Göttingen).

Incident locations were obtained from routinely documented prehospital scene location data and geocoded individually to generate case-specific coordinates. Road travel times were estimated using the Open Source Routing Machine (OSRM), based on OpenStreetMap data (https://www.openstreetmap.org/copyright), with a 25% reduction applied to approximate blue-light driving. Where geocoded coordinates were unavailable, the registry-documented transport time was used (*n* = 29); for patients with neither source available, the median OSRM-derived transport time was imputed (*n* = 10). Data completeness for all time variables used in the complementary analysis is reported in Supplementary Table S3. No imputation was performed for core clinical eligibility variables. All missing values in core eligibility variables were classified as not meeting the respective criterion unless otherwise specified.

Sustained ROSC was defined as achieving ROSC at any time point during prehospital care and additionally being admitted to the hospital with ROSC. Patients that achieved ROSC at any time point but were not transported or were transported with ongoing documented CPR were not classified as sustained ROSC cases.

ROSC times were calculated from EMS arrival to sustained ROSC. Total prehospital time was defined as the sum of EMS response time, duration of on-scene treatment and (hypothetical or documented) transport time.

No-flow time was derived directly from the registry in which it is categorised into five distinct categories (“≤5 Min”, “>5 - 10 Min”, “>10 - 15 Min”, “>15 Min” and “unwitnessed/unknown”) (Supplementary Table S6).

### Geospatial modelling

To simulate different intra-arrest transport strategies, we modelled three hypothetical scenarios with transport initiated after either 10, 15, or 20 minutes of on-scene EMS time. This was done irrespective of the actual treatment strategy documented and follows an approach similar to Vos et al. [18]. Patients who had achieved sustained ROSC before the modelled transport decision time were excluded. Patients were considered eligible for eCPR if they had not achieved sustained ROSC before hospital arrival, or if ROSC occurred only after the calculated hospital arrival time. Additionally, the total prehospital time had to be 60 minutes or less. Only patients arriving at the eCPR centre within this time window without ROSC were considered eCPR candidates.

For each transport scenario, the number of patients meeting eCPR eligibility criteria was calculated, alongside the proportion of the initial cohort this represented and the reasons for exclusion (e.g., early ROSC, prehospital time > 60 min, or missing data).

### Graphical representation of patient states and ROSC timing

Proportions of patients in each state are shown to visualise the distribution of patient states (i.e. ROSC before time point T, ROSC before hospital arrival, eCPR-eligible at hospital arrival, “uncertain” if unknown ROSC timing, and arrival at hospital > 60 min) at each T during the resuscitation phase. Patients are classified into mutually exclusive states at each decision point T. Where a patient with unknown ROSC timing also exceeds the 60-minute threshold, the patient is classified as “>60 minutes” rather than “uncertain”, as the time constraint alone precludes eCPR eligibility regardless of ROSC status. Additionally, a graphical analysis of time from EMS arrival to sustained ROSC was conducted to describe the temporal distribution of sustained ROSC relative to EMS arrival.

### Primary study endpoint

The primary endpoint was the proportion of patients meeting eCPR eligibility criteria at initial on-scene assessment and upon hypothetical hospital arrival in each of the three modelled transport scenarios. To estimate eCPR eligibility for the full regional OHCA cohort (*n* = 2,218), documented case counts were scaled proportionally based on the ratio of total OHCA cases to registry-documented cases (2,218/1,696), assuming a similar distribution of eligibility characteristics among undocumented cases.

### Procedural time analysis for hypothetical eCPR initiation

Because hospital arrival does not correspond to initiation of extracorporeal flow, we performed a complementary procedural time analysis estimating time from collapse to hypothetical eCPR initiation.

This analysis extended the transport-based modelling approach of Vos et al. [18], and was based on the time-interval framework described by Erblich et al. [21]. It was evaluated against the ELSO-recommended target of establishing extracorporeal flow within 60 minutes of cardiac arrest [20].

For each patient eligible at baseline, two hypothetical scenarios (S1 and S2) were modelled. The on-scene eCPR scenario (S1) was further split into parts *a* and *b*. For S1a time from collapse to eCPR initiation was defined as the sum of a hypothetical prehospital eCPR team’s response time (had it been dispatched simultaneously with the regular EMS crew) plus 24 minutes for on-scene initiation of eCPR. For S1b time until eCPR initiation was defined as the time from collapse until arrival of the dedicated eCPR team had it been dispatched after 10 minutes of on-scene assessment by the primary EMS crew plus 24 minutes for on-scene initiation of eCPR.

In the in-hospital eCPR scenario (S2), time from collapse to eCPR initiation was defined as the sum of the first EMS (ambulance or physician) response time, 15 minutes of advanced life support on-scene, 15 minutes for transport preparation under ongoing CPR, the individual transport time to the nearest eCPR-capable centre, five minutes for transfer to the emergency department, and 22 minutes for eCPR initiation in the hospital. The origin of all procedural time constants is detailed in Supplementary Table S1.

All scenarios were evaluated against a 60-minute threshold from collapse to hypothetical eCPR initiation. Whether sustained ROSC had occurred before the scenario- and case-specific hypothetical eCPR initiation time point was assessed. Patients with documented ROSC before that time were classified as no longer requiring eCPR. This allowed estimation of the proportion of patients who would both still require and could theoretically receive eCPR within the recommended low-flow window. Because ROSC timing was derived from the observed clinical course, this analysis should be interpreted as an approximation, as ROSC timing may differ under alternative treatment strategies.

### Use of large language models

The authors used ChatGPT-5 (OpenAI) and Claude Opus 4.8 (Anthropic) to assist with phrasing, language refinement, and troubleshooting of programming code. All AI-assisted text and code suggestions were reviewed, verified, and revised by the authors, who assume full responsibility for the accuracy of the final manuscript.

### Statistical analysis

This is a descriptive modelling study; no confirmatory hypotheses were tested. Continuous variables are summarised as median [interquartile range, IQR] and, for modelled times to eCPR, additionally as mean; categorical variables as counts (percentages). Eligibility counts and scenario endpoints are reported as absolute numbers and proportions of the analysed cohorts. All analyses were performed in Python within a *Quarto* notebook, geospatial processing used *geopandas* and *shapely* and driving times were obtained from the *Open Source Routing Machine* (OSRM).

## Results

### Cohort description

During the study period a total of 2,218 OHCA cases were documented within the regional EMS database, of which 1,696 cases with attempted resuscitation were documented in the local German Resuscitation Registry. Given the population of approximately 328,000 inhabitants in the city and county of Göttingen, this corresponds to an estimated annual incidence of ~ 67 OHCA cases per 100,000 inhabitants, which is in line with recent national data [22].

The analysed cohort (*n* = 1,696) had a median age of 71.5 years (IQR 59–81), and 67.5% were male. Most arrests occurred in a domestic environment (58.3%) and were witnessed in 55.7% of cases (42.8% by bystanders, 11.7% by EMS personnel and 1.2% by first responders). Bystander CPR was performed in 49.7%, and dispatcher-assisted CPR was documented in 26.8% of all cases.

The initial rhythm was shockable (ventricular fibrillation (VF)/ventricular tachycardia (VT)) in 20.5%, pulseless electrical activity (PEA) in 23.9%, and asystole in 55.3% of cases. A presumed cardiac cause was identified in 61.3%. Detailed ROSC status (including time to ROSC) was known in almost all cases, with 66.2% not achieving sustained ROSC during prehospital care. Further details can be found in Tables 1 and 2.

**Table 1:** Baseline characteristics—local eCPR protocol (eligible vs. non-eligible at baseline)

		Missing	Overall	eCPR-eligible	Non-eligible
n			1696	348	1348
Age (years), median [Q1, Q3]		14	71.5 [59.0,81.0]	63.5 [57.0,70.0]	76.0 [61.0,83.0]
Sex, n (%)	Female		552 (32.5)	82 (23.6)	470 (34.9)
	Male		1144 (67.5)	266 (76.4)	878 (65.1)
Location of arrest, n (%)	Home		989 (58.3)	183 (52.6)	806 (59.8)
	Public		707 (41.7)	165 (47.4)	542 (40.2)
Arrest witnessed by, n (%)	Bystander		726 (42.8)	246 (70.7)	480 (35.6)
	EMS (paramedic)		80 (4.7)	39 (11.2)	41 (3.0)
	EMS (physician)		118 (7.0)	53 (15.2)	65 (4.8)
	First Responder		21 (1.2)	10 (2.9)	11 (0.8)
	Unwitnessed		751 (44.3)	0 (0.0)	751 (55.7)
Bystander CPR, n (%)	No		853 (50.3)	153 (44.0)	700 (51.9)
	Yes		843 (49.7)	195 (56.0)	648 (48.1)
Dispatcher-assisted CPR, n (%)	No		1241 (73.2)	235 (67.5)	1006 (74.6)
	Yes		455 (26.8)	113 (32.5)	342 (25.4)
EMS response time (min), median [Q1, Q3]		22	9.0 [6.0,13.0]	7.5 [3.0,11.0]	10.0 [6.0,14.0]
Initial rhythm, n (%)	Asystole		938 (55.3)	113 (32.5)	825 (61.2)
	PEA		406 (23.9)	85 (24.4)	321 (23.8)
	VF/VT		347 (20.5)	150 (43.1)	197 (14.6)
	Other/unknown		5 (0.3)	0 (0.0)	5 (0.4)
Defibrillation count, n (%)	No defibrillation		1205 (71.0)	166 (47.7)	1039 (77.1)
	1 Shock		155 (9.1)	51 (14.7)	104 (7.7)
	Up to 3		167 (9.8)	66 (19.0)	101 (7.5)
	4 to 6		108 (6.4)	48 (13.8)	60 (4.5)
	7 to 9		41 (2.4)	6 (1.7)	35 (2.6)
	More than 9		20 (1.2)	11 (3.2)	9 (0.7)
Suspected aetiology, n (%)	Cardiac		1039 (61.3)	246 (70.7)	793 (58.8)
	Hypoxia		294 (17.3)	69 (19.8)	225 (16.7)
	Drowning		12 (0.7)	0 (0.0)	12 (0.9)
	Other or unknown		253 (14.9)	33 (9.5)	220 (16.3)
	Trauma		98 (5.8)	0 (0.0)	98 (7.3)
ROSC status, n (%)	No sustained ROSC		1122 (66.2)	149 (42.8)	973 (72.2)
	Sustained ROSC - time unknown		57 (3.4)	15 (4.3)	42 (3.1)
	Sustained ROSC during EMS treatment		494 (29.1)	172 (49.4)	322 (23.9)
	Sustained ROSC prior to EMS arrival		23 (1.4)	12 (3.4)	11 (0.8)
Prehospital outcome, n (%)	Admission with ROSC		574 (33.8)	199 (57.2)	375 (27.8)
	Death at scene		942 (55.5)	83 (23.9)	859 (63.7)
	Transport under CPR		180 (10.6)	66 (19.0)	114 (8.5)
Documented transport time (min), median [Q1, Q3]		992	13.0 [9.0,20.0]	14.0 [9.0,20.0]	13.0 [8.0,20.0]
Calculated transport time to eCPR centre (min), median [Q1, Q3]		1348	9.3 [7.0,17.3]	9.3 [7.0,17.3]	N/A

**Table 2:** Baseline characteristics—modified ELSO criteria (eligible vs. non-eligible at baseline)

		Missing	Overall	eCPR-eligible	Non-eligible
n			1696	149	1547
Age (years), median [Q1, Q3]		14	71.5 [59.0,81.0]	60.0 [54.0,65.0]	74.0 [60.0,82.0]
Sex, n (%)	Female		552 (32.5)	24 (16.1)	528 (34.1)
	Male		1144 (67.5)	125 (83.9)	1019 (65.9)
Location of arrest, n (%)	Home		989 (58.3)	67 (45.0)	922 (59.6)
	Public		707 (41.7)	82 (55.0)	625 (40.4)
Arrest witnessed by, n (%)	Bystander		726 (42.8)	102 (68.5)	624 (40.3)
	EMS (paramedic)		80 (4.7)	18 (12.1)	62 (4.0)
	EMS (physician)		118 (7.0)	26 (17.4)	92 (5.9)
	First Responder		21 (1.2)	3 (2.0)	18 (1.2)
	Unwitnessed		751 (44.3)	0 (0.0)	751 (48.5)
Bystander CPR, n (%)	No		853 (50.3)	61 (40.9)	792 (51.2)
	Yes		843 (49.7)	88 (59.1)	755 (48.8)
Dispatcher-assisted CPR, n (%)	No		1241 (73.2)	98 (65.8)	1143 (73.9)
	Yes		455 (26.8)	51 (34.2)	404 (26.1)
EMS response time (min), median [Q1, Q3]		22	9.0 [6.0,13.0]	7.0 [1.8,10.0]	9.0 [6.0,13.8]
Initial rhythm, n (%)	PEA		406 (23.9)	55 (36.9)	351 (22.7)
	VF/VT		347 (20.5)	94 (63.1)	253 (16.4)
	Asystole		938 (55.3)	0 (0.0)	938 (60.6)
	Other/unknown		5 (0.3)	0 (0.0)	5 (0.3)
Defibrillation count, n (%)	No defibrillation		1205 (71.0)	48 (32.2)	1157 (74.8)
	1 Shock		155 (9.1)	35 (23.5)	120 (7.8)
	Up to 3		167 (9.8)	27 (18.1)	140 (9.0)
	4 to 6		108 (6.4)	33 (22.1)	75 (4.8)
	7 to 9		41 (2.4)	1 (0.7)	40 (2.6)
	More than 9		20 (1.2)	5 (3.4)	15 (1.0)
Suspected aetiology, n (%)	Cardiac		1039 (61.3)	119 (79.9)	920 (59.5)
	Hypoxia		294 (17.3)	19 (12.8)	275 (17.8)
	Drowning		12 (0.7)	0 (0.0)	12 (0.8)
	Other or unknown		253 (14.9)	11 (7.3)	242 (15.6)
	Trauma		98 (5.8)	0 (0.0)	98 (6.3)
ROSC status, n (%)	No sustained ROSC		1122 (66.2)	48 (32.2)	1074 (69.4)
	Sustained ROSC - time unknown		57 (3.4)	7 (4.7)	50 (3.2)
	Sustained ROSC during EMS treatment		494 (29.1)	88 (59.1)	406 (26.2)
	Sustained ROSC prior to EMS arrival		23 (1.4)	6 (4.0)	17 (1.1)
Prehospital outcome, n (%)	Admission with ROSC		574 (33.8)	101 (67.8)	473 (30.6)
	Death at scene		942 (55.5)	20 (13.4)	922 (59.6)
	Transport under CPR		180 (10.6)	28 (18.8)	152 (9.8)
Documented transport time (min), median [Q1, Q3]		992	13.0 [9.0,20.0]	15.0 [10.0,20.0]	13.0 [8.0,20.0]
Calculated transport time to eCPR centre (min), median [Q1, Q3]		1348	9.3 [7.0,17.3]	10.0 [7.0,18.7]	9.2 [7.1,16.6]

### Eligibility for eCPR

To define the baseline eCPR eligibility cohort at EMS arrival, we applied stepwise exclusion criteria. Under the local protocol, patients were excluded if aged > 75 years or aged < 16 years, had a no-flow interval > 10 minutes or unknown, had a traumatic aetiology, or the arrest was unwitnessed. This resulted in a cohort of 348 patients (20.5% of all documented cases) meeting all predefined clinical criteria for baseline eCPR eligibility (Fig. 1).

Under the modified ELSO criteria the following inclusion criteria were applied: age 16–69 years, no-flow ≤ 5 minutes, and an initial rhythm of VF/VT or PEA. This yielded 149 eligible patients (8.8% of all documented cases; Fig. 2).

### Comparative characteristics

Patients eligible for eCPR at baseline (under the local protocol) were considerably younger than those excluded (median age 63.5 vs. 76.0 years), more likely to present with a shockable rhythm, and more often had a cardiac cause of arrest. They were also more frequently witnessed by bystanders or EMS personnel and more often received bystander CPR.

### Graphical representation of patient state

**Fig. 3:**
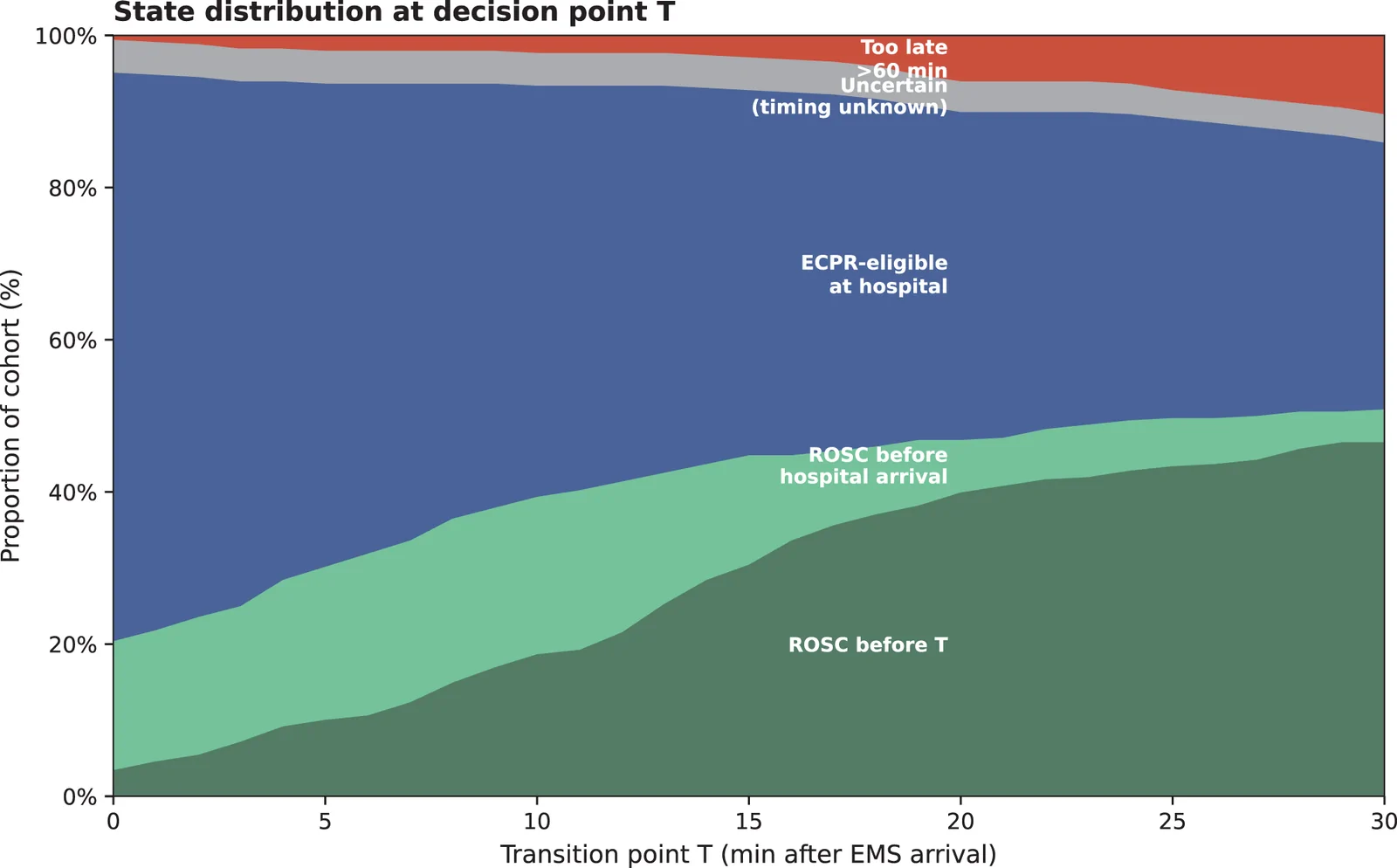
State distribution of the local-protocol cohort at each modelled decision point T (minutes after EMS arrival). The stacked areas show the proportion of patients with sustained ROSC before T, the proportion with sustained ROSC before hospital arrival, the proportion still eCPR-eligible at hospital arrival, the proportion with unknown ROSC timing, and the proportion for whom hospital arrival would occur after the 60-minute low-flow threshold. The figure illustrates how the composition of the potential eCPR pool changes with longer on-scene resuscitation

Figure 3 illustrates the shift in patient state distribution with increasing on-scene time. At *T* = 10 minutes, 18.7% of the cohort had already achieved sustained ROSC, 20.7% were projected to achieve ROSC during transport before arrival at the hospital, and 54.0% remained eCPR-eligible upon hospital arrival. By *T* = 20 minutes, the proportion with pre-transport ROSC had risen to 39.9%, while eCPR eligibility at hospital arrival decreased to 43.1%, and 6.0% exceeded the 60-minute time window. Throughout all time points, 3.7–4.3% of patients remained in the “uncertain” category due to unknown ROSC timing. This analysis was only conducted for the local cohort.

### ROSC dynamics

**Fig. 4:**
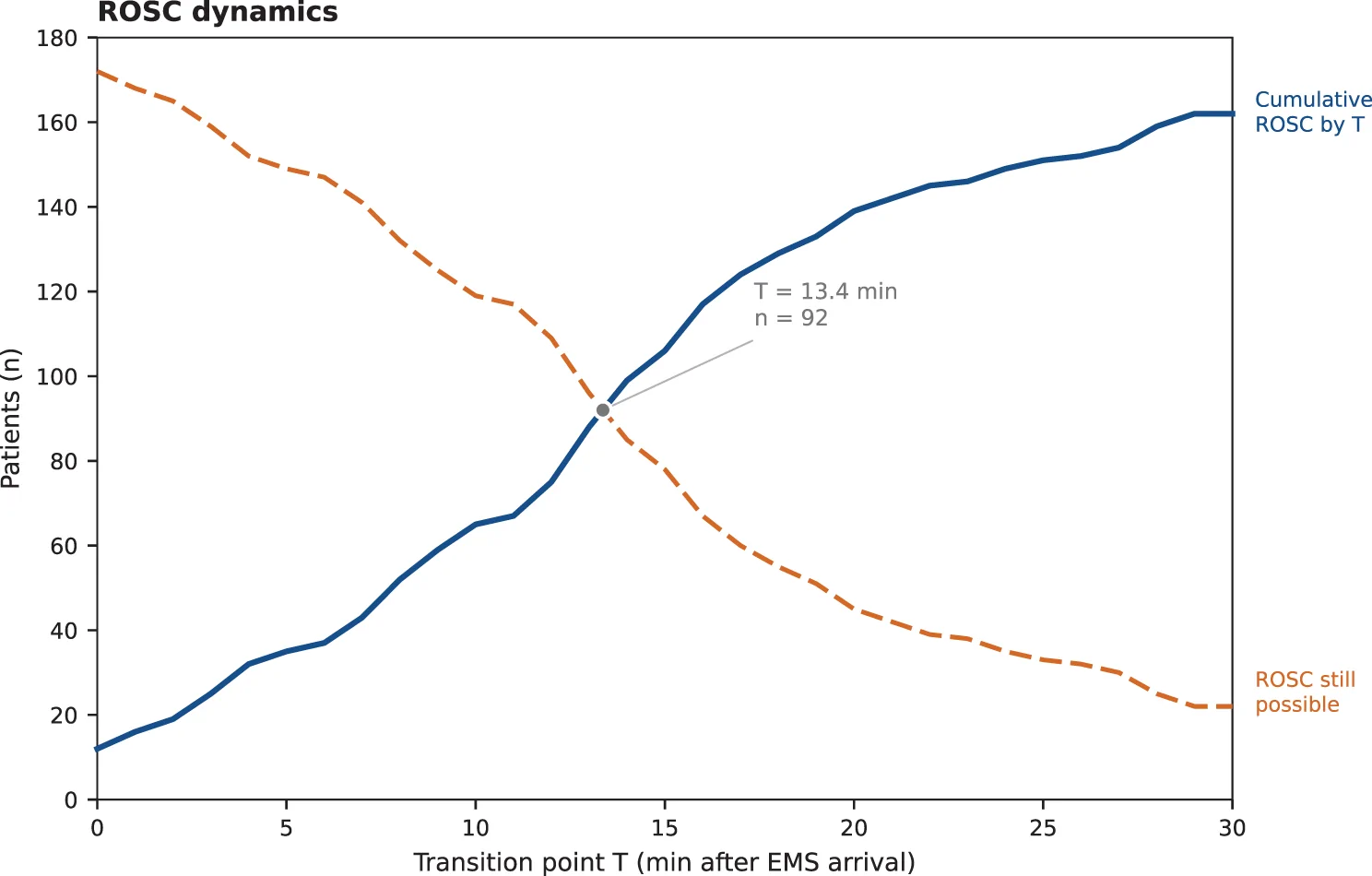
Cumulative ROSC dynamics by on-scene CPR duration in the first 30 minutes after EMS arrival, restricted to patients with sustained ROSC. Solid blue line = number of patients with sustained ROSC before decision point T; dashed orange line = number of sustained-ROSC patients whose ROSC occurred after T. The marker shows the crossover point where cumulative ROSC exceeds the remaining sustained-ROSC pool

Among patients with documented sustained ROSC, time from EMS arrival to sustained ROSC was analysed to describe when sustained ROSC accrued over time. This transition point was defined as the time at which the cumulative number of patients with documented sustained ROSC exceeded the number of patients without documented sustained ROSC up to that time point. The curves intersected at 13.4 minutes after EMS arrival, corresponding to 92 patients in each group (Fig. 4).

### eCPR eligibility at hospital arrival

**Fig. 5:**
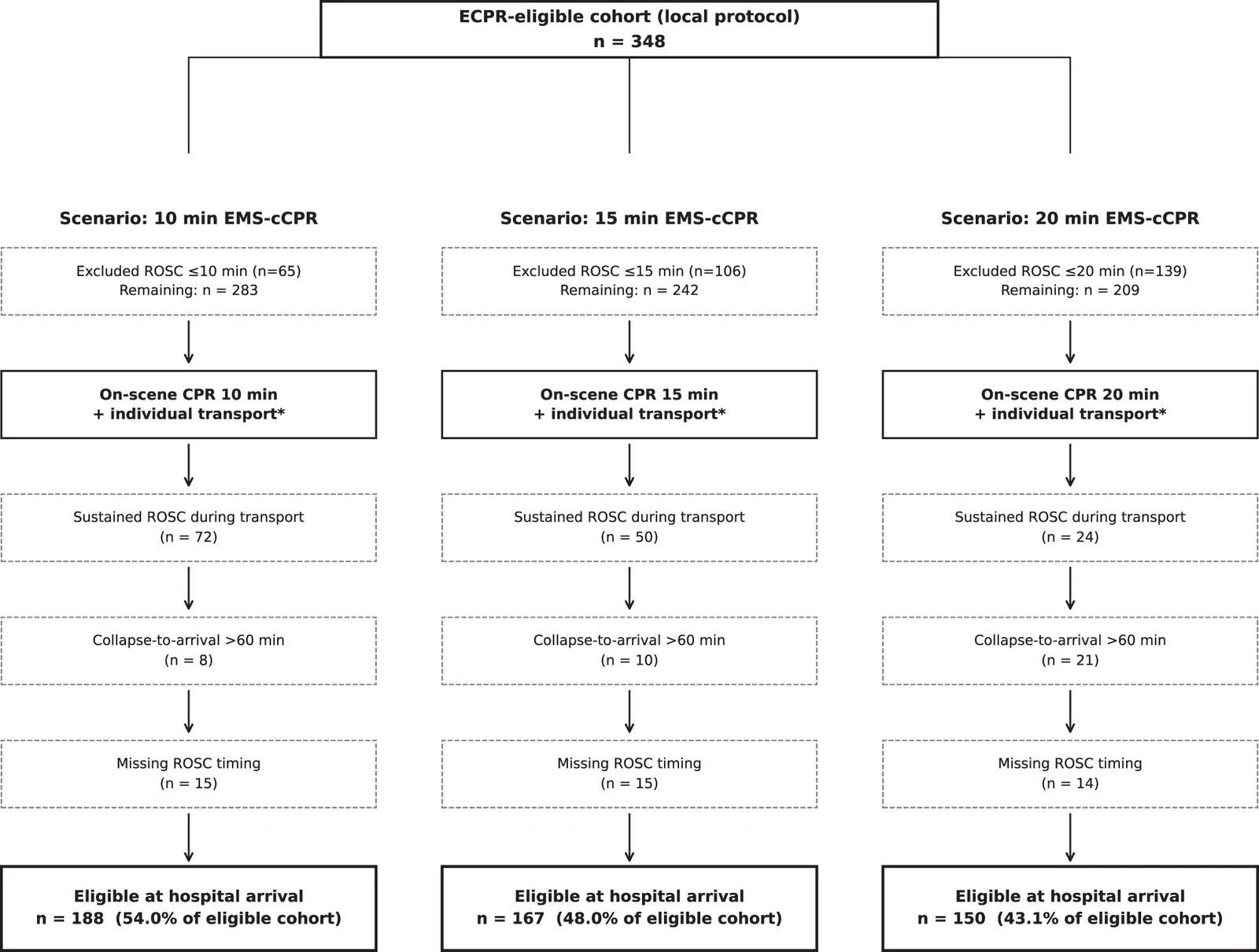
eCPR eligibility at hospital arrival - three on-scene CPR duration scenarios (local protocol)

**Fig. 6:**
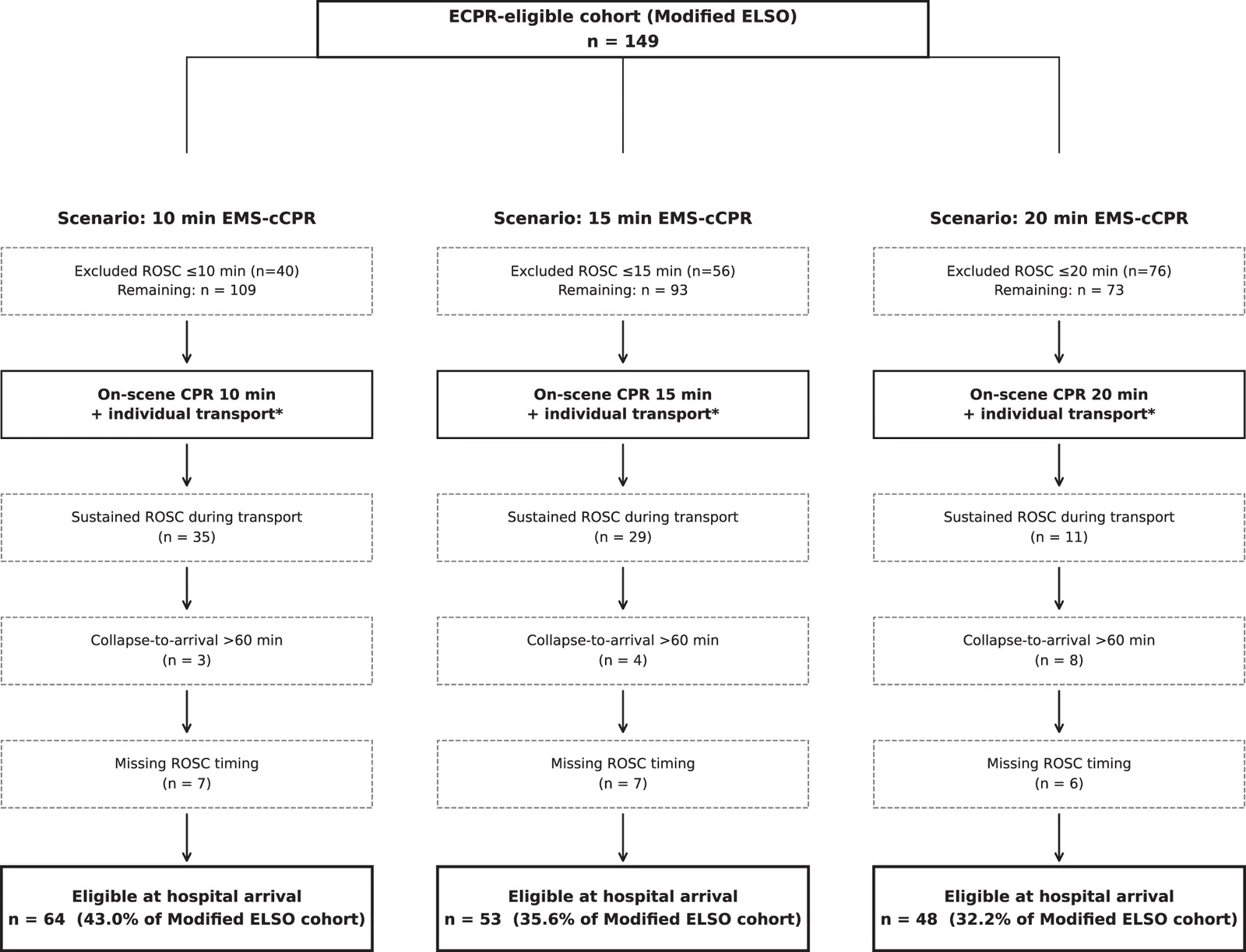
eCPR eligibility at hospital arrival - three on-scene CPR duration scenarios (modified ELSO criteria)

Depending on the assumed duration of on-scene resuscitation (*T* = 10, 15, and 20 minutes), 188 (54.0%), 167 (48.0%), and 150 (43.1%) patients, respectively, would have met the inclusion criteria of the local protocol (Fig. 5). Analogously, under the modified ELSO criteria, 64 (43.0%), 53 (35.6%), and 48 (32.2%) patients would have been eligible at hospital arrival (Fig. 6).

### Extrapolation to the regional OHCA population

Extrapolating by the ratio of total to documented cases (2,218/1,696 = 1.31), we estimated approximately 455 potentially eCPR-eligible patients under the local protocol over the 10-year study period at EMS arrival, corresponding to approximately 46 patients per year. Using the modified ELSO criteria, the extrapolated baseline estimate was approximately 195 patients (≈20 per year).

Depending on the timing of intra-arrest transport initiation, the estimated number of patients still meeting local eCPR eligibility upon hospital arrival would have been 246 (11.1%) after 10 minutes, 218 (9.8%) after 15 minutes, and 196 (8.8%) after 20 minutes of on-scene CPR (Table 3) - if transport had been started at these time points. This corresponds to an estimated number of 20–25 cases per year who would arrive at the hospital still eligible for in-hospital eCPR initiation under the local protocol. Considering the cohort derived from the modified ELSO criteria these numbers amounted to 195 (8.8%) at baseline and 84 (3.8%), 69 (3.1%) and 63 (2.8%) had transport been started after 10, 15 and 20 minutes, respectively.

**Table 3:** Extrapolation from documented (*n* = 1,696) to full cohort (*n* = 2,218)

	Documented	Extrapolated	% of cohort
All OHCA (documented)	1,696	2,218	100.0%
**Local protocol**			
Eligible at baseline	348	455	20.5%
Eligible at hospital (*T* = 10 min)	188	246	11.1%
Eligible at hospital (*T* = 15 min)	167	218	9.8%
Eligible at hospital (*T* = 20 min)	150	196	8.8%
**Modified ELSO criteria**			
Eligible at baseline	149	195	8.8%
Eligible at hospital (*T* = 10 min)	64	84	3.8%
Eligible at hospital (*T* = 15 min)	53	69	3.1%
Eligible at hospital (*T* = 20 min)	48	63	2.8%

### On-scene versus in-hospital hypothetical eCPR initiation

The median estimated time from collapse to eCPR in the on-scene scenario with simultaneous dispatch of the eCPR team (S1a) was 37 minutes (IQR 34–45) for the local protocol cohort and 37 minutes (IQR 34–46) for the modified ELSO cohort, with 94.8% and 97.3% of patients falling within the 60-minute low-flow window (Supplementary Table S4, Supplementary Figure S2). For scenario S1b estimated time to eCPR initiation was 54 minutes (IQR 49–62) and 54 minutes (IQR 49–61) in the local cohort and modified ELSO cohort, respectively, with 71.6% and 73.2% potentially receiving eCPR within 60 minutes. In the in-hospital scenario (S2), median times were 75 minutes (IQR 70–83; local cohort) and 75 minutes (IQR 70–82; modified ELSO), with fewer than 1% reaching eCPR initiation within 60 minutes (Supplementary Figure S2). After excluding patients who achieved sustained ROSC before the hypothetical eCPR initiation time point, 47.4% (165/348; S1a) of local protocol and 36.9% (55/149; S1a) of modified ELSO patients and 31.6% (110/348; S1b) and 24.8% (37/149; S1b) would both still have required and could have received on-scene eCPR within 60 minutes (Supplementary Table S4, Supplementary Figure S3). For in-hospital eCPR, this applied to one patient in each cohort.

## Discussion

In this retrospective temporospatial analysis of OHCA in a mixed urban-rural German region, we combined two complementary modelling approaches to estimate regional eCPR feasibility. First, following a transport-based approach, we assessed eCPR eligibility at EMS arrival and upon hypothetical hospital arrival after intra-arrest transport. Second, we estimated whether eCPR could realistically be initiated within the recommended 60-minute low-flow window.

The transport-based analysis showed that approximately one in five patients met local eCPR eligibility criteria at EMS arrival, while fewer than one in ten fulfilled the stricter modified ELSO criteria. Eligibility upon hypothetical hospital arrival decreased with increasing on-scene resuscitation time, mainly because many patients achieved sustained ROSC before arrival.

The procedural time analysis demonstrated that hospital arrival did not translate into timely eCPR initiation. Estimated median time to eCPR was 37 and 54 minutes for the on-scene scenarios (S1a and S1b respectively), compared with 75 minutes after intra-arrest transport for in-hospital eCPR. Thus, eCPR initiation would theoretically have been feasible within 60 minutes for a majority of all patients in the on-scene scenario, but for fewer than 1% in the in-hospital scenario.

These findings indicate that feasibility analyses based on hospital arrival alone may substantially overestimate the practical feasibility of in-hospital eCPR after intra-arrest transport.

### Baseline eCPR eligibility in the context of existing literature

Our finding that approximately one in five EMS-treated OHCA patients met local eCPR eligibility criteria aligns closely with recent analyses from comparable settings. Erblich et al. analysed 6,687 non-traumatic OHCA patients in Austria over a decade and found that 13.3% fulfilled eCPR eligibility criteria at EMS arrival using guideline-based criteria (age 12–70, witnessed, bystander/EMS CPR, shockable rhythm or PEA, no ROSC) [21]. In a sensitivity analysis including all cardiac rhythms, 21.6% were eligible; a figure that closely mirrors our own 20.5% under the local protocol. Their sensitivity analysis restricted to shockable rhythms yielded 11.2%, comparable to our 8.8% under modified ELSO criteria.

In contrast, Vos et al. (2023) conducted a similar geospatial modelling study in the northern Netherlands but applied more restrictive criteria, specifically requiring immediate bystander CPR, an initial shockable rhythm, and witnessed arrest, resulting in a smaller eligible cohort [18]. Gottula et al. performed geographic information system-based (GIS) analyses in the United States and reported variable eligibility rates depending on criteria applied and geographic access to eCPR centres [23, 24].

These differences highlight that direct comparison of eligibility rates across studies is limited by heterogeneous inclusion criteria.

### The role of initial cardiac rhythm in eCPR eligibility

The inclusion of non-shockable rhythms may raise questions, as these patients represent a subgroup with particularly poor outcomes. However, several lines of evidence support consideration of selected non-shockable patients for eCPR:

Kawauchi et al. developed a clinical prediction score specifically for eCPR in patients with initial non-shockable rhythms, suggesting that within this group, a subset with favourable features can be identified [15].

Lunz et al. reported that 12.2% of eCPR survivors initially presented with PEA and 1.4% with asystole—demonstrating that survival is possible even with non-shockable rhythms when patients are appropriately selected [25]. However, the same study identified initial asystole as the strongest independent on-scene predictor of poor outcome (OR 35.0).

A meta-analysis by Taccone et al. found that eCPR benefit was most pronounced in patients with non-shockable rhythms combined with non-cardiac cause of arrest—a counterintuitive finding that merits further investigation. It should be noted, however, that overall survival in this patient group was very low and the patients in this subgroup might have been highly selected cases [26].

The inclusion of patients with non-shockable rhythms remains controversial; however, within this subgroup, patients presenting with PEA are often considered the most plausible candidates to benefit from eCPR. Importantly, our findings should not be interpreted as evidence of benefit of eCPR in patients with non-shockable rhythms; instead, they indicate that exclusion of this subgroup substantially alters the size of the potentially eligible population. In the present analysis, inclusion of PEA primarily affects estimated programme volume and should not be interpreted as evidence that such patients derive comparable benefit from eCPR.

### ROSC dynamics and the evolving eligibility pool

As expected, sustained ROSC was the most frequent reason why patients no longer fulfilled eCPR eligibility criteria over time. Earlier transport may preserve a larger pool of patients eligible for eCPR, whereas patients with early ROSC may not require eCPR. This cross-over point describes the temporal distribution of sustained ROSC but does not by itself define an optimal transport time; a patient-centred threshold would require a decision analysis weighing survival outcomes, which is beyond the scope of this study.

It should be considered that cCPR quality may deteriorate during transport compared with on-scene resuscitation. Conversely, delaying transport to maintain higher-quality cCPR may prolong time to eCPR if sustained ROSC is not achieved.

Our findings resonate with the INCEPTION trial experience, where a substantial proportion of patients randomised to eCPR achieved ROSC before ECMO flow was established—illustrating the challenge of defining “refractory” cardiac arrest in real time [27].

### Hospital arrival ≠ eCPR initiation—the procedural time gap

Although our primary analysis used hospital arrival as the reference point, this does not correspond to the start of extracorporeal blood flow. In the complementary in-hospital eCPR scenario (S2), median estimated time from collapse to eCPR initiation was 75 minutes in both the local cohort and the modified ELSO cohort. Accordingly, only 0.6% and 0.7% of patients, respectively, would have remained within the 60-minute threshold. In contrast, the on-scene eCPR scenario (S1a) yielded a median time of 37 minutes, with >90% of patients remaining within 60 minutes. In-hospital eCPR may systematically exceed the 60-minute threshold in settings comparable to our region, despite being logistically easier to implement.

Erblich et al. reached very similar conclusions, reinforcing the view that in-hospital eCPR after intra-arrest transport is unlikely to meet the 60-minute target in routine practice [21].

A recent simulation study by Leroux et al. suggested that prehospital eCPR is associated with markedly shorter low-flow times and a higher likelihood of achieving ECMO flow within 60 minutes than hospital-based strategies [28]. However, published prehospital eCPR programmes also underline the major logistical demands of this strategy and the relatively small proportion of screened patients who ultimately undergo cannulation [25].

This should not be used as a simple argument in favour of on-scene eCPR. Our retrospective data cannot account for the effect of team experience on cannulation success and outcomes. In the current system, in-hospital cannulation at high-volume eCPR centres may represent the more realistic, and possibly preferable, strategy.

Assuming the door-to-ECLS times of a high-volume centre with intra-arrest transport (Prague OHCA trial, median 12 min) [29], in-hospital eCPR within 60 minutes would become feasible for approximately half of eligible patients (Supplementary Table S7). Still, modelled on-scene initiation remained superior, suggesting that our comparison of strategies is robust to assumptions about in-hospital performance.

### Regional eCPR planning—case volumes and resource allocation

Sufficient case volume is crucial for maintaining team competency and procedural quality in eCPR. The Regensburg programme performed 213 on-scene eCPR procedures over 10 years and reported outcomes comparable to or better than those of the randomised trials, suggesting that the case volumes estimated under our local protocol may be sufficient to support a viable eCPR programme [25]. Our temporospatial modelling provides a data-driven basis for the planning of such programmes. It has to be taken into account that only a subset of the patients that would theoretically have been eligible in our retrospective analysis would have still required eCPR after exclusions for end-tidal CO2, comorbidity, team availability, or competing emergencies.

## Limitations

This study has several limitations. First, its retrospective design and reliance on registry data introduce the risk of documentation errors, missing values, and variable data quality across EMS providers. Second, important metabolic variables relevant to real-world eCPR decision-making (e.g. pH, lactate, pO2, end-tidal CO2) were not available in the registry. Had these been available, the true number of eligible patients would likely have been lower. Third, no data were available on actual eCPR cannulations during the study period, and survival or neurological outcomes were not assessed. The analysis therefore addresses theoretical eligibility rather than real-world programme performance or treatment benefit. Fourth, the analysis regarding on-scene or in-hospital cannulation was based on published procedural time intervals from other centres rather than local data. Although absolute times may differ across systems, the marked difference between scenarios suggests that the overall conclusions are reasonable. Furthermore, our extrapolation from registry-documented cases to the full regional OHCA population assumed that undocumented cases had a similar distribution of eCPR eligibility characteristics, which may not have been the case and could have led to over- or underestimation of the true case volume.

Moreover, re-arrest times after transient ROSC are not documented in the registry; patients with transient ROSC were therefore classified as continuously requiring eCPR in the primary analysis, which might have overestimated eCPR eligibility. A sensitivity analysis using the documented timing of all ROSC episodes was conducted (Supplementary Table S8) and did not change the conclusions.

Additionally, the no-flow interval was undocumented in 40.5% of cases; in a best-case analysis treating all unknown intervals as fulfilling the criterion, baseline eligibility changed from 20.5% to 24.9% (local protocol) and 8.8% to 10.6% (modified ELSO) within the primary analysis, leaving all conclusions unchanged.

Finally, generalisability is limited by the single-region design and the physician-staffed, mixed urban-rural German EMS setting.

## Conclusions

Our temporospatial modelling provides a data-driven basis for regional eCPR planning and demonstrates that a substantial number of theoretically eligible patients exists in a mixed urban-rural German EMS region both on-scene and after transport. When realistic procedural time intervals were incorporated, in-hospital eCPR was unlikely to meet the 60-minute target, whereas on-scene eCPR remained theoretically feasible in most cases. These findings may inform regional planning of eCPR programmes and highlight the importance of including procedural time estimates in future feasibility analyses.

## Electronic supplementary material

Below is the link to the electronic supplementary material.

Supplementary material 1

Supplementary material 2

Supplementary material 3

Supplementary material 4

Supplementary material 5

Supplementary material 6

Supplementary material 7

## Data Availability

The datasets generated and/or analysed during the current study contain sensitive patient information and are therefore not publicly available. Access may be considered on reasonable request, subject to applicable ethical, legal, and institutional approvals.

## Abbreviations

cCPR: Conventional cardiopulmonary resuscitation
ECMO: Extracorporeal membrane oxygenation
eCPR: Extracorporeal cardiopulmonary resuscitation
ELSO: Extracorporeal Life Support Organization
EMS: Emergency medical services
GIS: Geographic information system
HEMS: Helicopter emergency medical services
OHCA: Out-of-hospital cardiac arrest
OSRM: Open Source Routing Machine
PEA: Pulseless electrical activity
ROSC: Return of spontaneous circulation
VA-ECMO: Veno-arterial extracorporeal membrane oxygenation
VF: Ventricular fibrillation
VT: Ventricular tachycardia
