# Supplementary material for: Modelling eCPR eligibility after out-of-hospital cardiac arrest: a 10-year retrospective temporospatial analysis from a mixed urban-rural region in Germany
Source: Scand J Trauma Resusc Emerg Med. 2026 Sep 9;34:159. doi: 10.1186/s13049-026-01681-7 (PMC13628803; doi:10.1186/s13049-026-01681-7)
Supplement: Supplementary file 1 [file 13049_2026_1681_MOESM1_ESM.pdf]

Modelling eCPR Eligibility After Out-of-Hospital Cardiac Arrest: A 10-Year Retrospective Temporospatial Analysis from a Mixed Urban-Rural Region in Germany

Clemens Grimm, Hanna Illian, Nils Kunze-Szikszay, Hanna Haus, Claudius Balzer, Markus Roessler

Main Results

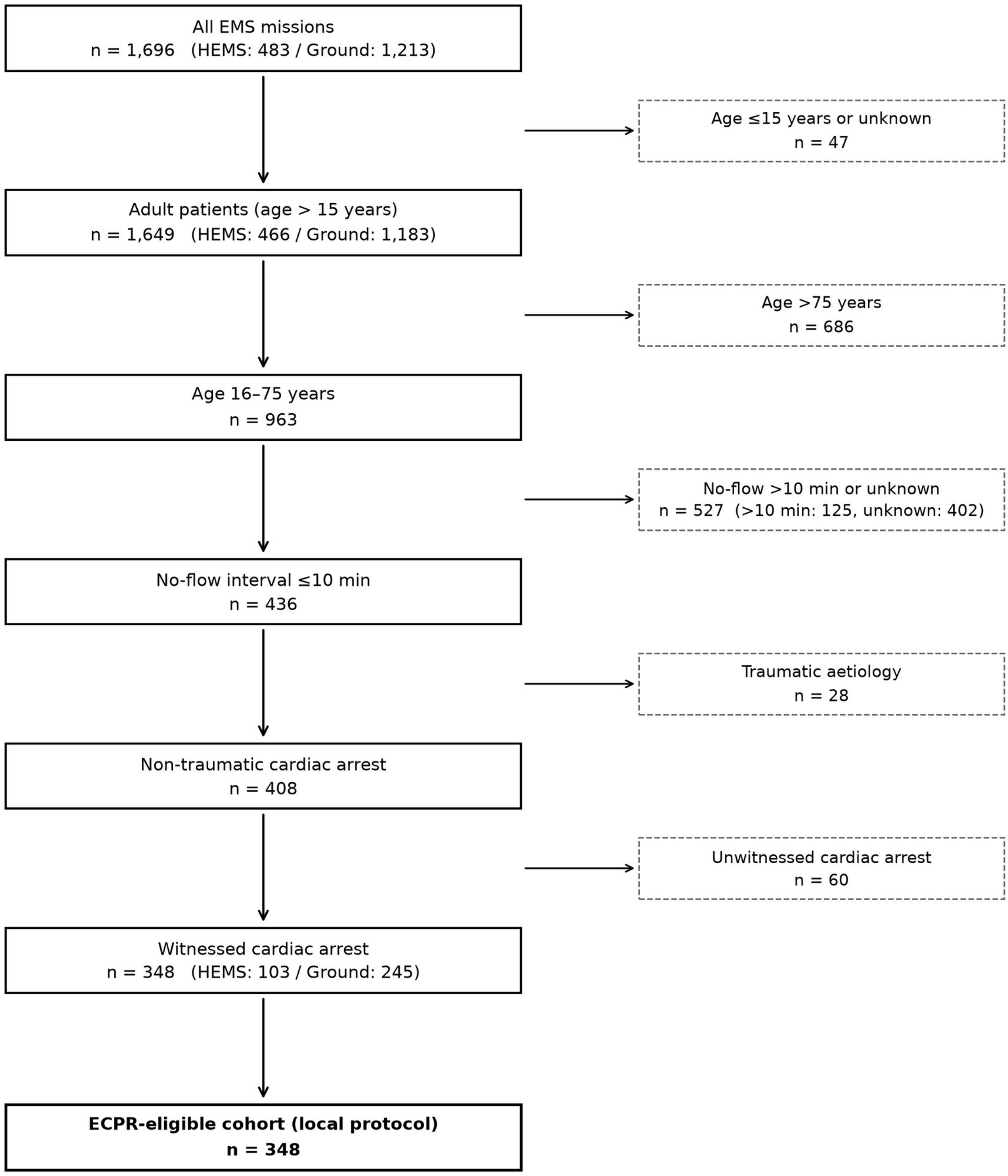

Figure 1 *Patient selection — local eCPR protocol.*

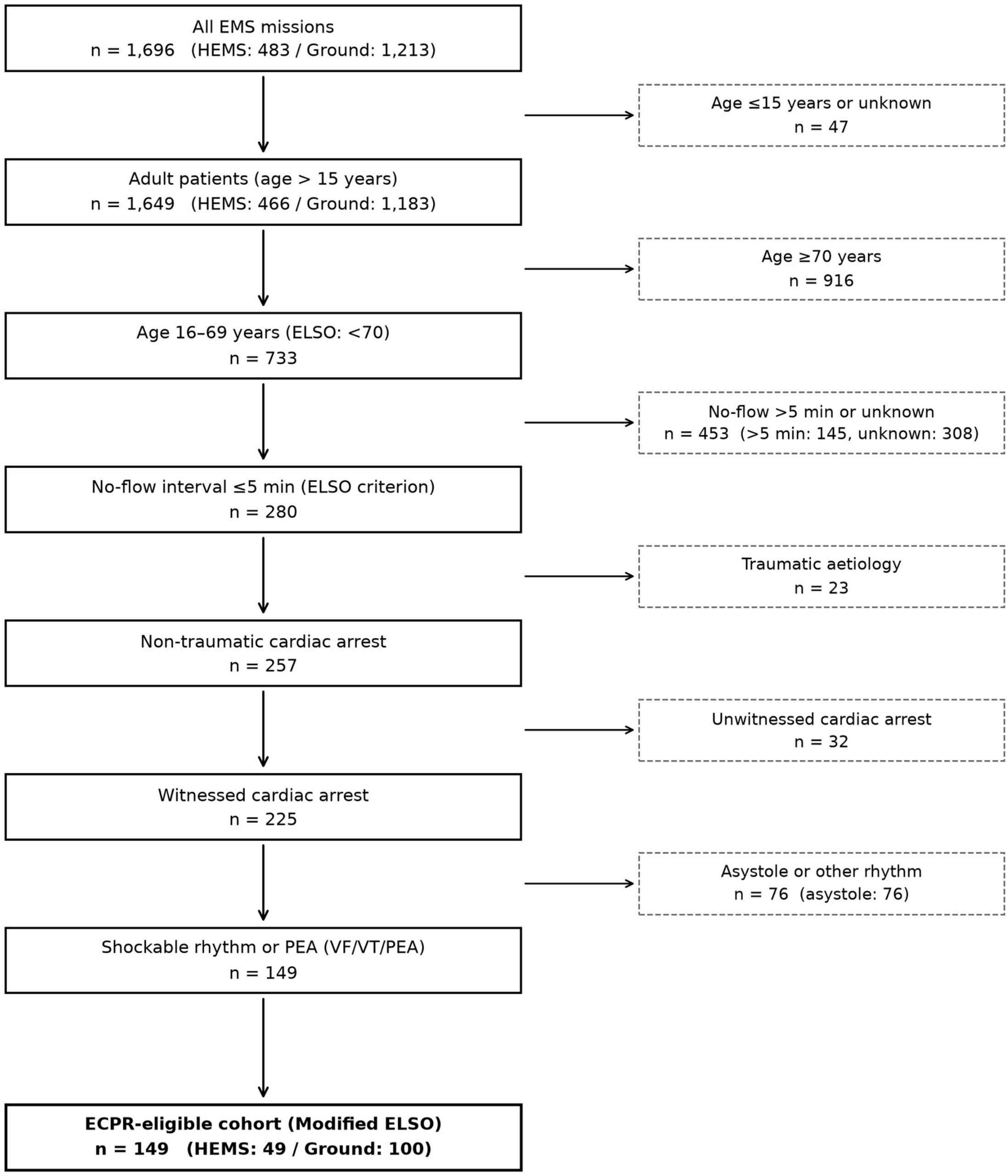

Figure 2 *Patient selection — modified ELSO cohort.*

Table 1 *Baseline characteristics — local eCPR protocol (eligible vs. non-eligible at baseline)*

		Missing	Overall	eCPR-eligible	Non-eligible
n			1696	348	1348
Age (years), median [Q1, Q3]		14	71.5 [59.0,81.0]	63.5 [57.0,70.0]	76.0 [61.0,83.0]
Sex, n (%)	Female		552 (32.5)	82 (23.6)	470 (34.9)
	Male		1144 (67.5)	266 (76.4)	878 (65.1)
Location of arrest, n (%)	Home		989 (58.3)	183 (52.6)	806 (59.8)
	Public		707 (41.7)	165 (47.4)	542 (40.2)
Arrest witnessed by, n (%)	Bystander		726 (42.8)	246 (70.7)	480 (35.6)
	EMS (paramedic)		80 (4.7)	39 (11.2)	41 (3.0)
	EMS (physician)		118 (7.0)	53 (15.2)	65 (4.8)
	First Responder		21 (1.2)	10 (2.9)	11 (0.8)
	Unwitnessed		751 (44.3)	0 (0.0)	751 (55.7)
Bystander CPR, n (%)	No		853 (50.3)	153 (44.0)	700 (51.9)
	Yes		843 (49.7)	195 (56.0)	648 (48.1)
Dispatcher-assisted CPR, n (%)	No		1241 (73.2)	235 (67.5)	1006 (74.6)
	Yes		455 (26.8)	113 (32.5)	342 (25.4)
EMS response time (min), median [Q1, Q3]		22	9.0 [6.0,13.0]	7.5 [3.0,11.0]	10.0 [6.0,14.0]
Initial rhythm, n (%)	Asystole		938 (55.3)	113 (32.5)	825 (61.2)
	PEA		406 (23.9)	85 (24.4)	321 (23.8)
	VF/VT		347 (20.5)	150 (43.1)	197 (14.6)
	Other/unknown		5 (0.3)	0 (0.0)	5 (0.4)
Defibrillation count, n (%)	No defibrillation		1205 (71.0)	166 (47.7)	1039 (77.1)
	1 Shock		155 (9.1)	51 (14.7)	104 (7.7)
	Up to 3		167 (9.8)	66 (19.0)	101 (7.5)
	4 to 6		108 (6.4)	48 (13.8)	60 (4.5)
	7 to 9		41 (2.4)	6 (1.7)	35 (2.6)
	More than 9		20 (1.2)	11 (3.2)	9 (0.7)
Suspected aetiology, n (%)	Cardiac		1039 (61.3)	246 (70.7)	793 (58.8)
	Hypoxia		294 (17.3)	69 (19.8)	225 (16.7)
	Drowning		12 (0.7)	0 (0.0)	12 (0.9)
	Other or unknown		253 (14.9)	33 (9.5)	220 (16.3)
	Trauma		98 (5.8)	0 (0.0)	98 (7.3)
ROSC status, n (%)	No sustained ROSC		1122 (66.2)	149 (42.8)	973 (72.2)

	sustained ROSC - time unknown		57 (3.4)	15 (4.3)	42 (3.1)
	sustained ROSC during EMS treatment		494 (29.1)	172 (49.4)	322 (23.9)
	sustained ROSC prior to EMS arrival		23 (1.4)	12 (3.4)	11 (0.8)
Prehospital outcome, n (%)	Admission with ROSC		574 (33.8)	199 (57.2)	375 (27.8)
	Death at scene		942 (55.5)	83 (23.9)	859 (63.7)
	Transport under CPR		180 (10.6)	66 (19.0)	114 (8.5)
Documented transport time (min), median [Q1, Q3]		992	13.0 [9.0,20.0]	14.0 [9.0,20.0]	13.0 [8.0,20.0]
Calculated transport time to eCPR centre (min), median [Q1, Q3]		1348	9.3 [7.0,17.3]	9.3 [7.0,17.3]	N/A

Table 2 *Baseline characteristics — modified ELSO criteria (eligible vs. non-eligible at baseline)*

		**Missing**	**Overall**	**eCPR-eligible**	**Non-eligible**
n			1696	149	1547
Age (years), median [Q1, Q3]		14	71.5 [59.0,81.0]	60.0 [54.0,65.0]	74.0 [60.0,82.0]
Sex, n (%)	Female		552 (32.5)	24 (16.1)	528 (34.1)
	Male		1144 (67.5)	125 (83.9)	1019 (65.9)
Location of arrest, n (%)	Home		989 (58.3)	67 (45.0)	922 (59.6)
	Public		707 (41.7)	82 (55.0)	625 (40.4)
Arrest witnessed by, n (%)	Bystander		726 (42.8)	102 (68.5)	624 (40.3)
	EMS (paramedic)		80 (4.7)	18 (12.1)	62 (4.0)
	EMS (physician)		118 (7.0)	26 (17.4)	92 (5.9)
	First Responder		21 (1.2)	3 (2.0)	18 (1.2)
	Unwitnessed		751 (44.3)	0 (0.0)	751 (48.5)
Bystander CPR, n (%)	No		853 (50.3)	61 (40.9)	792 (51.2)
	Yes		843 (49.7)	88 (59.1)	755 (48.8)
Dispatcher-assisted CPR, n (%)	No		1241 (73.2)	98 (65.8)	1143 (73.9)
	Yes		455 (26.8)	51 (34.2)	404 (26.1)
EMS response time (min), median [Q1, Q3]		22	9.0 [6.0,13.0]	7.0 [1.8,10.0]	9.0 [6.0,13.8]
Initial rhythm, n (%)	PEA		406 (23.9)	55 (36.9)	351 (22.7)
	VF/VT		347 (20.5)	94 (63.1)	253 (16.4)
	Asystole		938 (55.3)	0 (0.0)	938 (60.6)
	Other/ unknown		5 (0.3)	0 (0.0)	5 (0.3)
Defibrillation count, n (%)	No defibrillation		1205 (71.0)	48 (32.2)	1157 (74.8)
	1 Shock		155 (9.1)	35 (23.5)	120 (7.8)
	Up to 3		167 (9.8)	27 (18.1)	140 (9.0)
	4 to 6		108 (6.4)	33 (22.1)	75 (4.8)
	7 to 9		41 (2.4)	1 (0.7)	40 (2.6)
	More than 9		20 (1.2)	5 (3.4)	15 (1.0)
Suspected aetiology, n (%)	Cardiac		1039 (61.3)	119 (79.9)	920 (59.5)
	Hypoxia		294 (17.3)	19 (12.8)	275 (17.8)
	Drowning		12 (0.7)	0 (0.0)	12 (0.8)
	Other or unknown		253 (14.9)	11 (7.3)	242 (15.6)

	Trauma		98 (5.8)	0 (0.0)	98 (6.3)
ROSC status, n (%)	No sustained ROSC		1122 (66.2)	48 (32.2)	1074 (69.4)
	sustained ROSC - time unknown		57 (3.4)	7 (4.7)	50 (3.2)
	sustained ROSC during EMS treatment		494 (29.1)	88 (59.1)	406 (26.2)
	sustained ROSC prior to EMS arrival		23 (1.4)	6 (4.0)	17 (1.1)
Prehospital outcome, n (%)	Admission with ROSC		574 (33.8)	101 (67.8)	473 (30.6)
	Death at scene		942 (55.5)	20 (13.4)	922 (59.6)
	Transport under CPR		180 (10.6)	28 (18.8)	152 (9.8)
Documented transport time (min), median [Q1, Q3]		992	13.0 [9.0,20.0]	15.0 [10.0,20.0]	13.0 [8.0,20.0]
Calculated transport time to eCPR centre (min), median [Q1, Q3]		1348	9.3 [7.0,17.3]	10.0 [7.0,18.7]	9.2 [7.1,16.6]

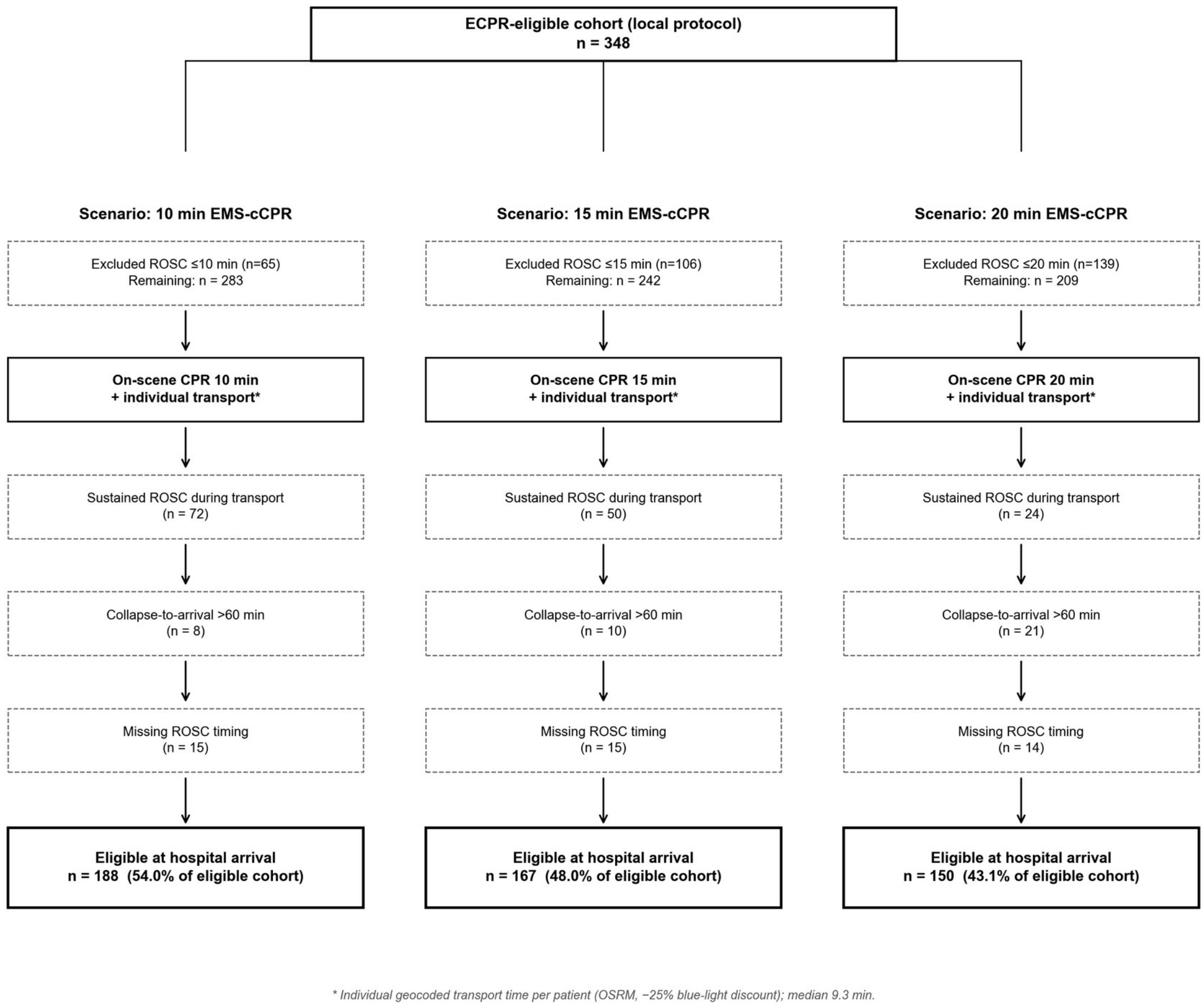

Figure 3 *eCPR eligibility at hospital arrival — three on-scene CPR duration scenarios (local protocol).*

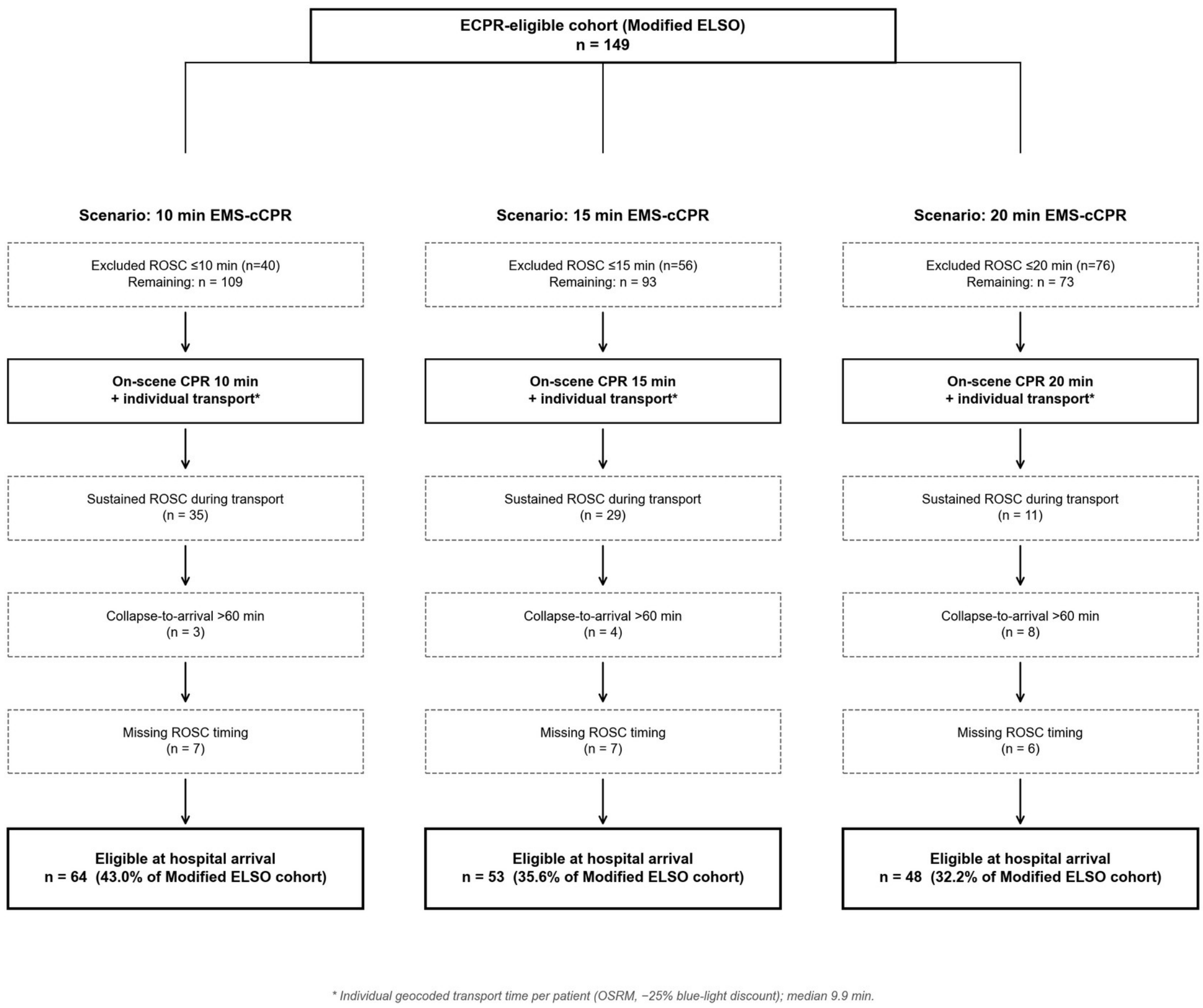

Figure 4 *eCPR eligibility at hospital arrival — three on-scene CPR duration scenarios (modified ELSO criteria).*

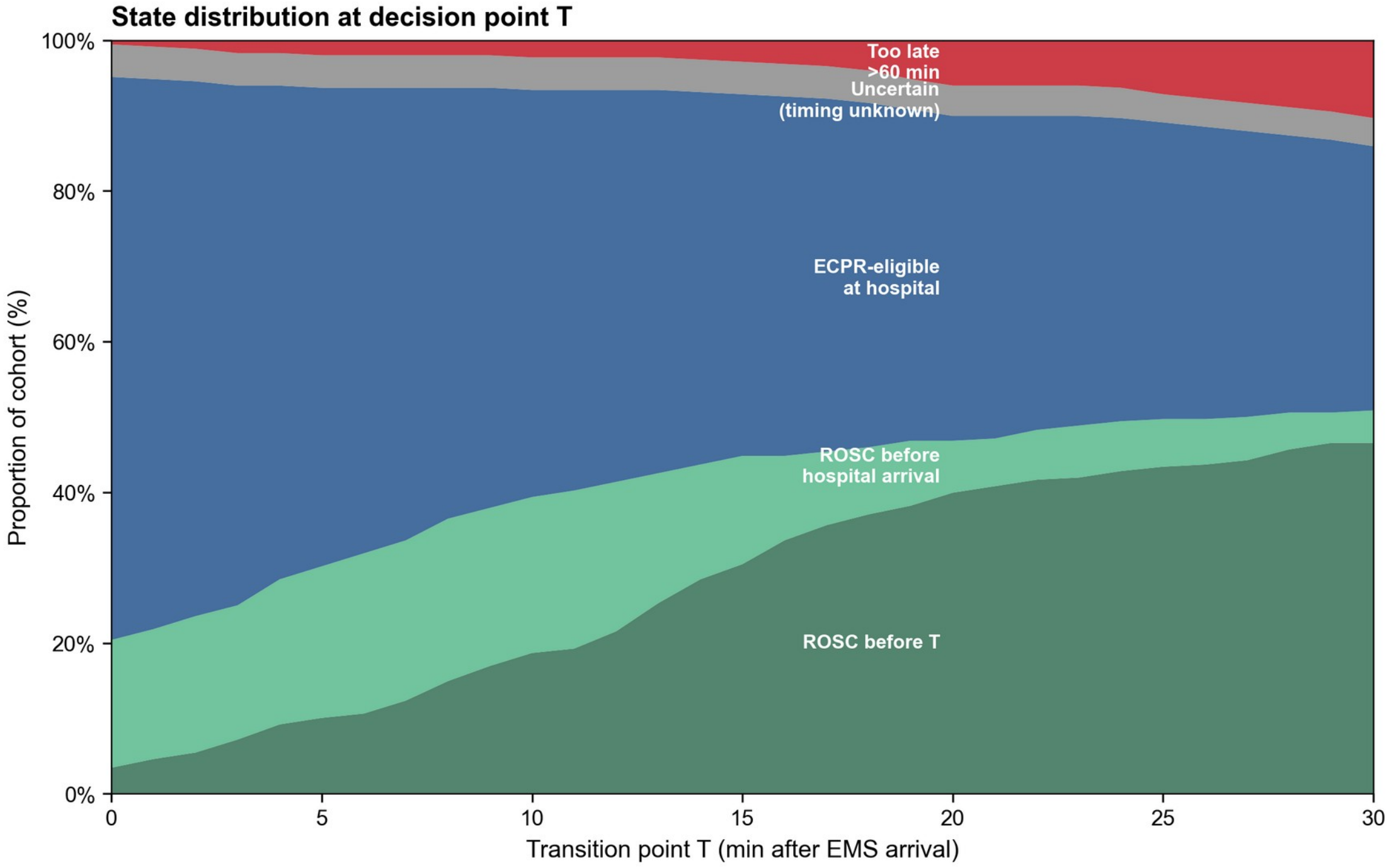

Figure 5 *State distribution of the local-protocol cohort at each modelled decision point T (minutes after EMS arrival). The stacked areas show the proportion of patients with sustained ROSC before T, the proportion with sustained ROSC before hospital arrival, the proportion still eCPR-eligible at hospital arrival, the proportion with unknown ROSC timing, and the proportion for whom hospital arrival would occur after the 60-minute low-flow threshold. The figure illustrates how the composition of the potential eCPR pool changes with longer on-scene resuscitation.*

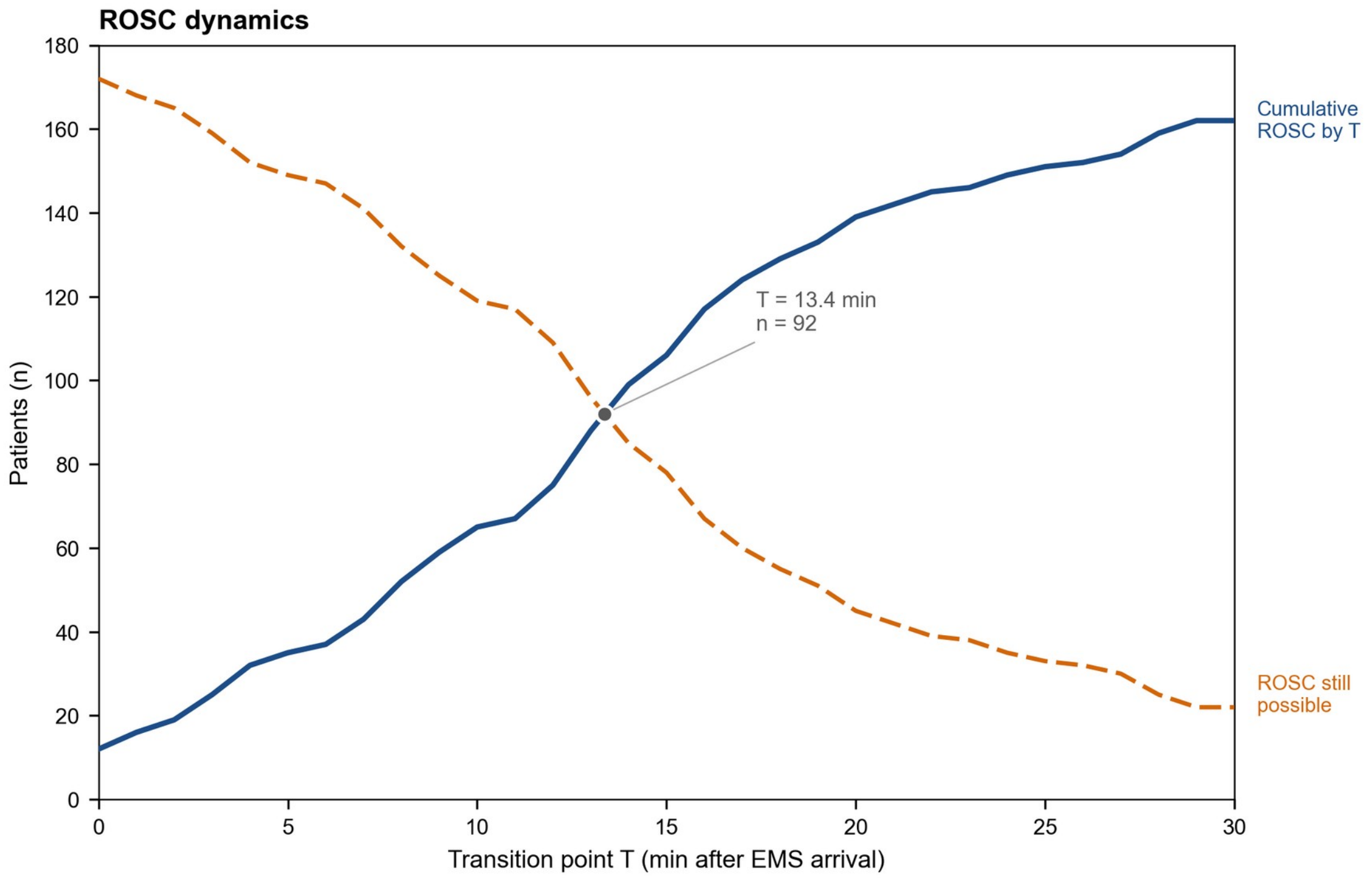

Figure 6 *Cumulative ROSC dynamics by on-scene CPR duration in the first 30 minutes after EMS arrival, restricted to patients with sustained ROSC. Solid blue line = number of patients with sustained ROSC before decision point T; dashed orange line = number of sustained-ROSC patients whose ROSC occurred after T. The marker shows the crossover point where cumulative ROSC exceeds the remaining sustained-ROSC pool.*

Table 3 *Extrapolation from documented (n = 1,696) to full cohort (n = 2,218)*

	**Documented**	**Extrapolated**	**% of cohort**
All OHCA (documented)	1,696	2,218	100.0%
**Local protocol**			
Eligible at baseline	348	455	20.5%
Eligible at hospital (T=10 min)	188	246	11.1%
Eligible at hospital (T=15 min)	167	218	9.8%
Eligible at hospital (T=20 min)	150	196	8.8%
**Modified ELSO criteria**			
Eligible at baseline	149	195	8.8%
Eligible at hospital (T=10 min)	64	84	3.8%
Eligible at hospital (T=15 min)	53	69	3.1%
Eligible at hospital (T=20 min)	48	63	2.8%

Supplementary material

Supplementary Table S1 *Time intervals for eCPR scenario modelling*

Component	Value	Scenario	Data source
Alarm delay (collapse to alarm)	Individual	S1a	Study registry (alarm minus collapse time; 0 where collapse time undocumented, cohort median where alarm missing)
eCPR team turnout	2 min	S1a and S1b	Model assumption (team staffed and ready, analogous to regular EMS units)
eCPR team travel to scene	Individual	S1a and S1b	OSRM road routing with blue-light discount factor 0.75 from eCPR centre to the OHCA location
First EMS response time (collapse to first EMS arrival)	Individual	S1b and S2	Study registry (earliest of paramedic or physician arrival minus collapse time)
Eligibility assessment by first EMS crew	10 min	S1b	Model assumption
On-scene eCPR cannulation and initiation	24 min	S1a and S1b	Erblich et al. (2025) [1]
Advanced life support on scene	15 min	S2	Vos et al. (2023) [2]; Grunau et al. (2016) [3]
Patient transport preparation and loading under ongoing CPR	15 min	S2	Erblich et al. (2025) [1]
Transport time to eCPR centre	Individual	S2	OSRM road routing with blue-light discount factor 0.75
Unloading and transfer to emergency department	5 min	S2	Erblich et al. (2025) [1]
eCPR initiation in emergency department	22 min	S2	Erblich et al. (2025) [1]

Supplementary Table S2 *Methodological comparison of primary analysis and procedural time scenarios*

**Component**	**Primary analysis**	**S1a (eCPR team dispatch at emergency call)**	**S1b (eCPR team dispatch after eligibility assessment)**	**S2 (in-hospital)**
Time zero	Collapse/Alarm	Collapse/Alarm	Collapse/Alarm	Collapse/Alarm
EMS arrival measured as	First EMS	—	First EMS	First EMS
Rationale	—	eCPR team co-dispatched at the emergency call	ECPR team alarmed after eligibility confirmed on scene	EMS can initiate advanced life support and transport
On-scene time	T = 10/15/20 min	24 min (eCPR setup)	24 min (eCPR setup)	ALS 15 min
Transport preparation under CPR	—	—	—	15 min
Transport	OSRM blue-light	—	—	OSRM blue-light
Unloading at hospital	—	—	—	5 min
eCPR setup and initiation in-hospital	—	—	—	22 min
**Endpoint**	**Hospital arrival**	**eCPR initiation**	**eCPR initiation**	**eCPR initiation**

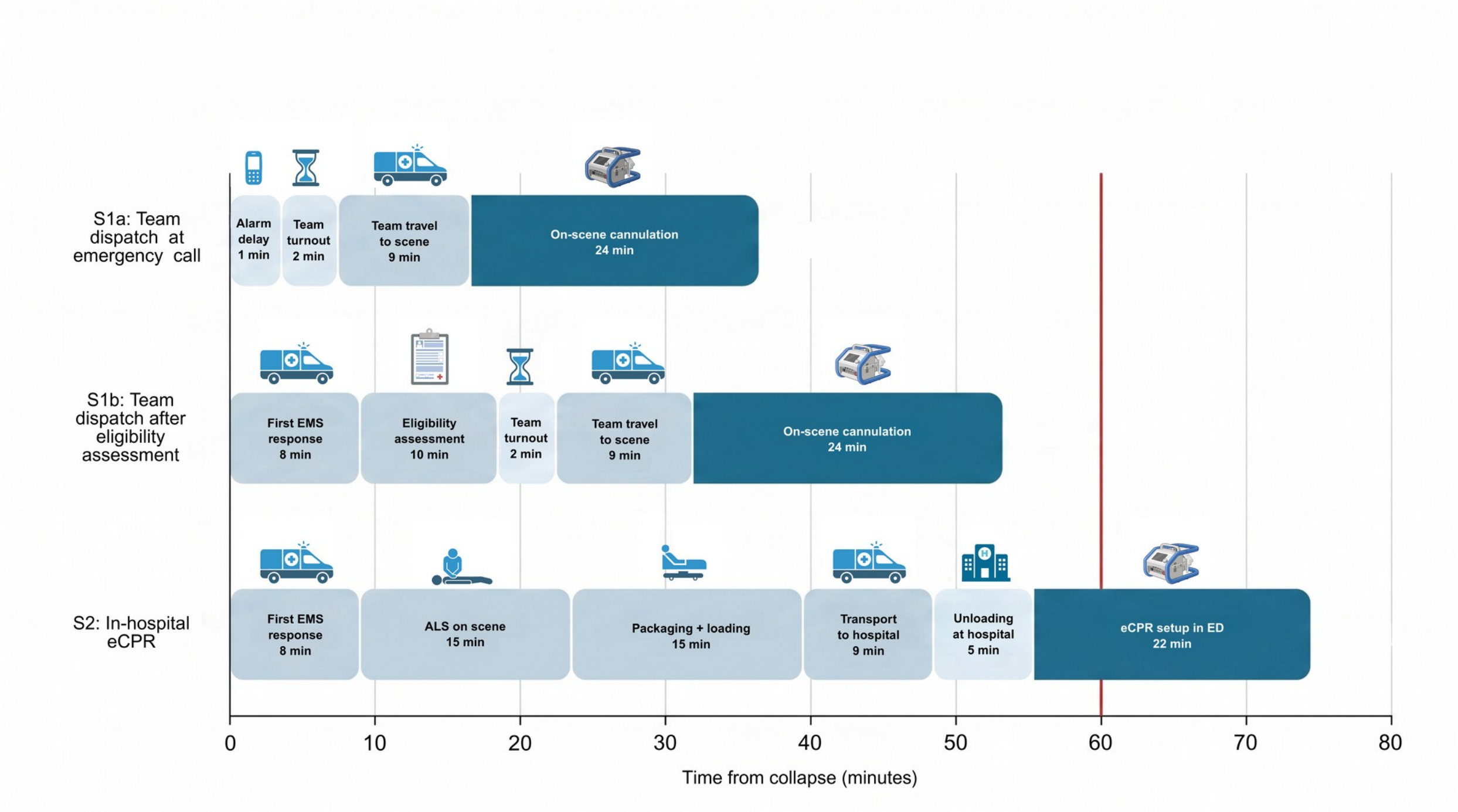

Supplementary Figure S1 *Cumulative time from collapse to hypothetical initiation of extracorporeal circulation across the three modelling scenarios. (Figure created in BioRender.com).*

Supplementary Table S3 *Data completeness for time variables*

	**Local protocol (n=348)**	**Modified ELSO (n=149)**
Physician response time	344 (98.9%) (missing: 4 (1.1%))	148 (99.3%) (missing: 1 (0.7%))
—Physician arrival available	343 (98.6%) (missing: 5 (1.4%))	147 (98.7%) (missing: 2 (1.3%))
—Collapse time available	332 (95.4%) (missing: 16 (4.6%))	142 (95.3%) (missing: 7 (4.7%))
Transport time to eCPR centre		
—OSRM road routing (blue-light)	309 (88.8%)	136 (91.3%)
—Registry-documented transport	29 (8.3%)	10 (6.7%)
—Cohort median imputation	10 (2.9%)	3 (2.0%)
ROSC timing (of sustained ROSC patients)	186/199 (93.5%) (missing: 13/199 (6.5%))	94/101 (93.1%) (missing: 7/101 (6.9%))

Supplementary Table S4 *Procedural time scenarios by cohort and calculation method*

**Cohort**	**Scenario**	**Mean ± SD (min)**	**Median [IQR] (min)**	**≤60 min n (%)**	**Still requiring eCPR AND ≤60 min n (%)**
**Local protocol (n = 348)**	S1a Team dispatch at emergency call	40.7 ± 9.7	37 [34-45]	330 (94.8%)	165 (47.4%)
	S1b Team dispatch after eligibility assessment	57.2 ± 12.7	54 [49-62]	249 (71.6%)	110 (31.6%)
	S2 In-hospital eCPR	78.2 ± 12.7	75 [70-83]	2 (0.6%)	1 (0.3%)
**Modified ELSO (n = 149)**	S1a Team dispatch at emergency call	40.7 ± 9.6	37 [34-46]	145 (97.3%)	55 (36.9%)
	S1b Team dispatch after eligibility assessment	56.6 ± 11.8	54 [49-61]	109 (73.2%)	37 (24.8%)
	S2 In-hospital eCPR	77.6 ± 11.8	75 [70-82]	1 (0.7%)	1 (0.7%)

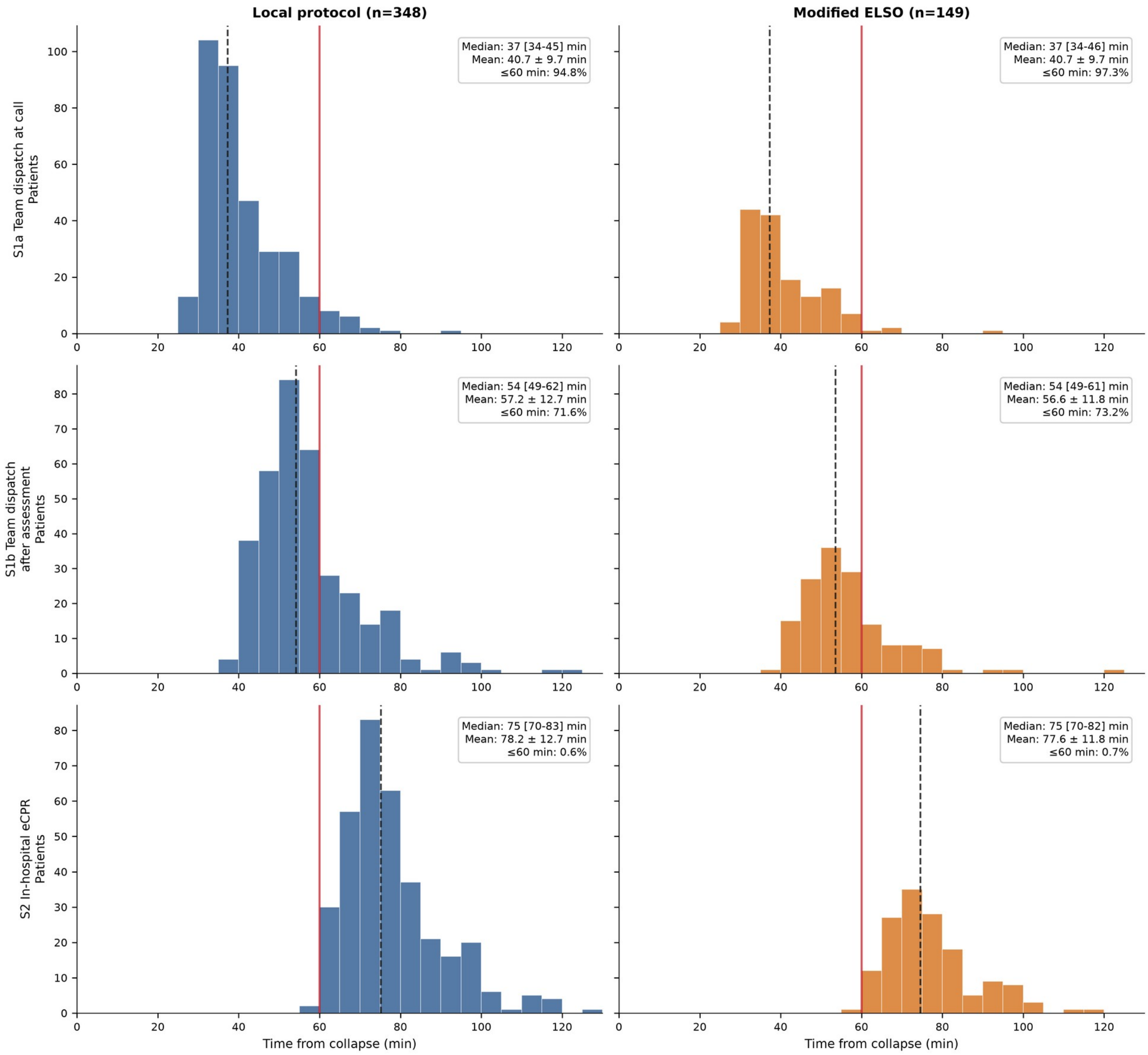

Supplementary Figure S2 *Distribution of estimated time from collapse to hypothetical eCPR initiation for local protocol and modified ELSO cohorts. Solid vertical line = 60-minute guideline. Dashed lines = medians.*

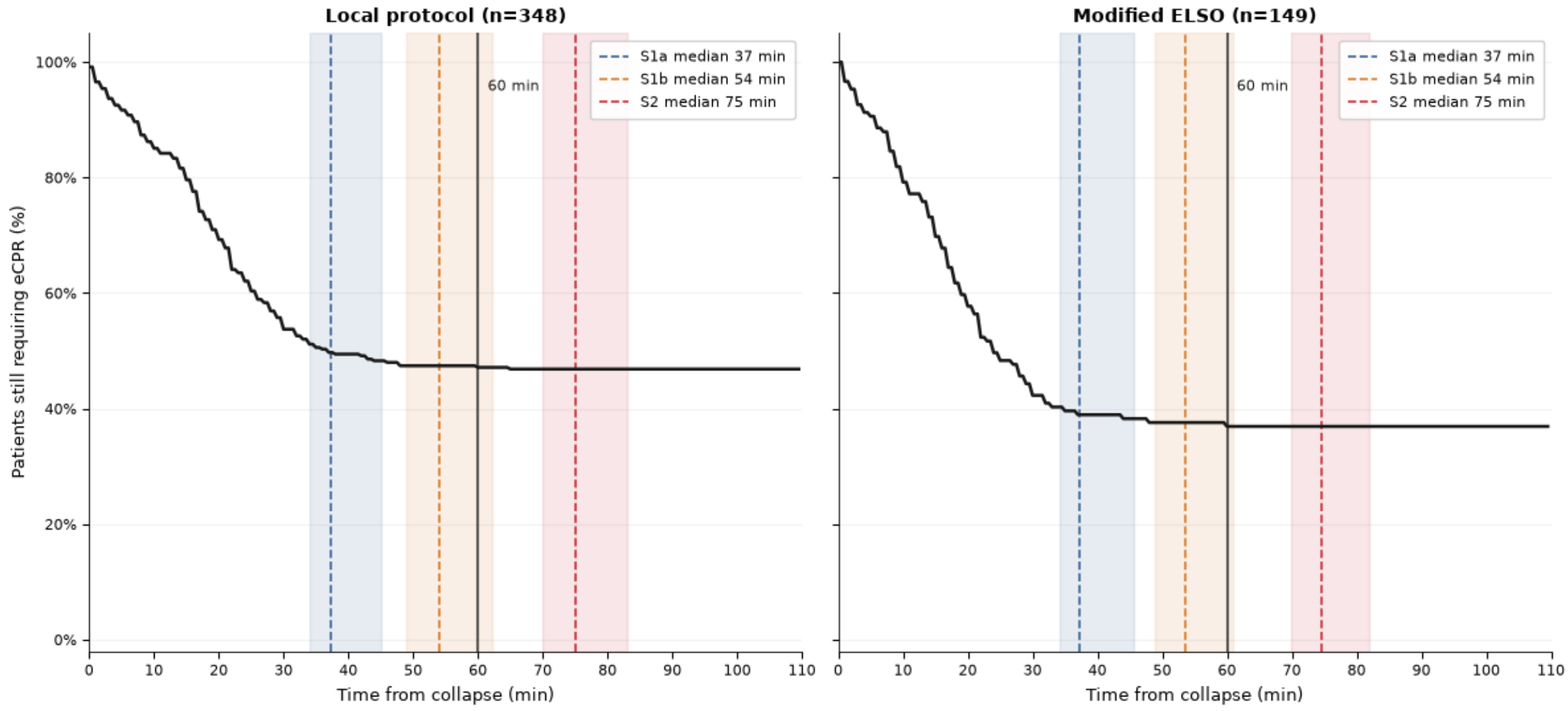

Supplementary Figure S3 *Proportion of patients without sustained ROSC (i.e. still requiring eCPR) over time from collapse. The curve falls as patients achieve ROSC. Shaded bands indicate the interquartile range (IQR) of estimated eCPR initiation times for scenarios S1a, S1b and scenario S2, with dashed lines at the median. The solid vertical line marks the recommended 60-minute low-flow time window.*

Supplementary Table S5 *Study setting characteristics*

Characteristic	
EMS district	Landkreis Göttingen
Geographic area	1,755 km²
Population served	~328,000 (187/km²)
Annual OHCA incidence	67.6 per 100,000 inhabitants
eCPR-capable centre	University Medical Centre Göttingen (UMG)
Urban / periurban / rural — share of area	3% / 10% / 87%
Urban / periurban / rural — share of population	53% / 38% / 10%
Geocoded eligible cases (local protocol)	309/348 (88.8%)
Urban / periurban / rural — share of eligible cases	52% / 33% / 15%
Driving time scene → UMG (OSRM, blue-light), median [IQR]	9.3 [6.9–17.5] min
Driving time scene → UMG, mean ± SD	13.4 ± 9.7 min
Eligible cases within 10 / 20 / 30 min of UMG	54% / 81% / 93%
District area within 10 / 20 / 30 min of UMG	4% / 26% / 54%

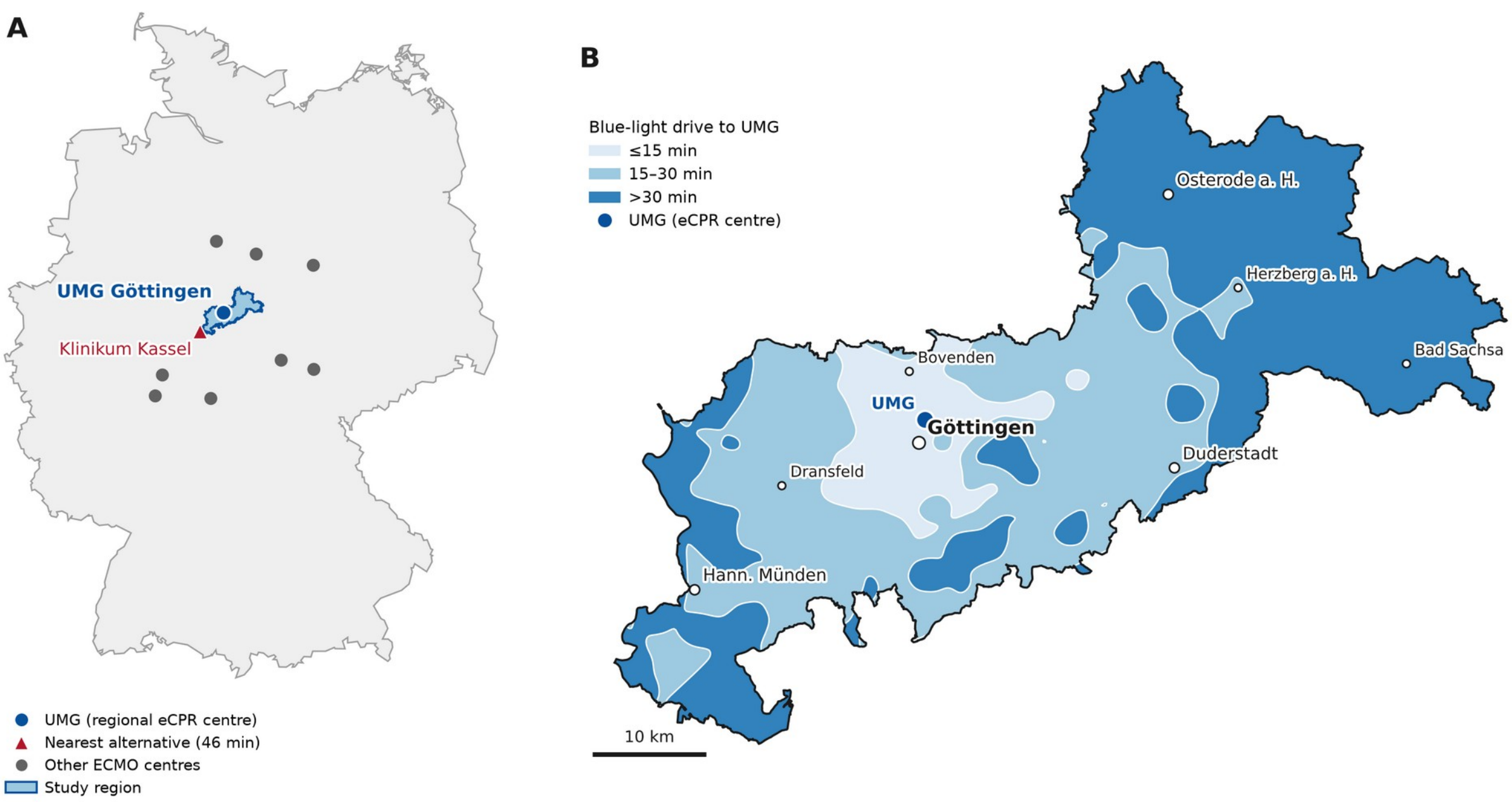

Supplementary Figure S4 *(A) Location of the study region within Germany and location of adjacent eCPR centres. (B) Blue-light drive times to the local eCPR centre (University Medical Centre Göttingen)*

Supplementary Table S6 *Distribution of documented no-flow intervals among cases excluded for exceeding the no-flow threshold. The median category is the category containing the median rank.*

Documented no-flow category	Modified ELSO: excluded for no-flow >5 min	Local protocol: excluded for no-flow >10 min
>5–10 min	51 (35.2%)	— (within threshold)
>10–15 min	43 (29.7%)	55 (44.0%)
>15 min	51 (35.2%)	70 (56.0%)
Total excluded	145	125
Median category	>10–15 min	>15 min

Supplementary Table S7 *Scenario S2 (transport with in-hospital eCPR) under Prague-OHCA in-hospital times [4]. The combined in-hospital interval (hospital arrival to extracorporeal flow; base case 5 + 22 = 27 min) is replaced by the door-to-eCPR time reported for the Prague OHCA trial (median 12 min, IQR 11–14) [4]. These time intervals are applied here as a best-case bound for the in-hospital segment of S2.*

**Cohort**	**Scenario**	**Mean ± SD (min)**	**Median [IQR] (min)**	**≤60 min n (%)**	**Still requiring eCPR AND ≤60 min n (%)**
**Local protocol (n = 348)**	S1a Team dispatch at emergency call	40.7 ± 9.7	37 [34-45]	330 (94.8%)	165 (47.4%)
	S1b Team dispatch after eligibility assessment	57.2 ± 12.7	54 [49-62]	249 (71.6%)	110 (31.6%)
	S2 base case: 27 min in-hospital	78.2 ± 12.7	75 [70-83]	2 (0.6%)	1 (0.3%)
	S2 Prague door-to-ECLS, median: 12 min	63.2 ± 12.7	60 [55-68]	172 (49.4%)	80 (23.0%)
**Modified ELSO (n = 149)**	S1a Team dispatch at emergency call	40.7 ± 9.6	37 [34-46]	145 (97.3%)	55 (36.9%)
	S1b Team dispatch after eligibility assessment	56.6 ± 11.8	54 [49-61]	109 (73.2%)	37 (24.8%)
	S2 base case: 27 min in-hospital	77.6 ± 11.8	75 [70-82]	1 (0.7%)	1 (0.7%)
	S2 Prague door-to-ECLS, median: 12 min	62.6 ± 11.8	60 [55-67]	75 (50.3%)	28 (18.8%)

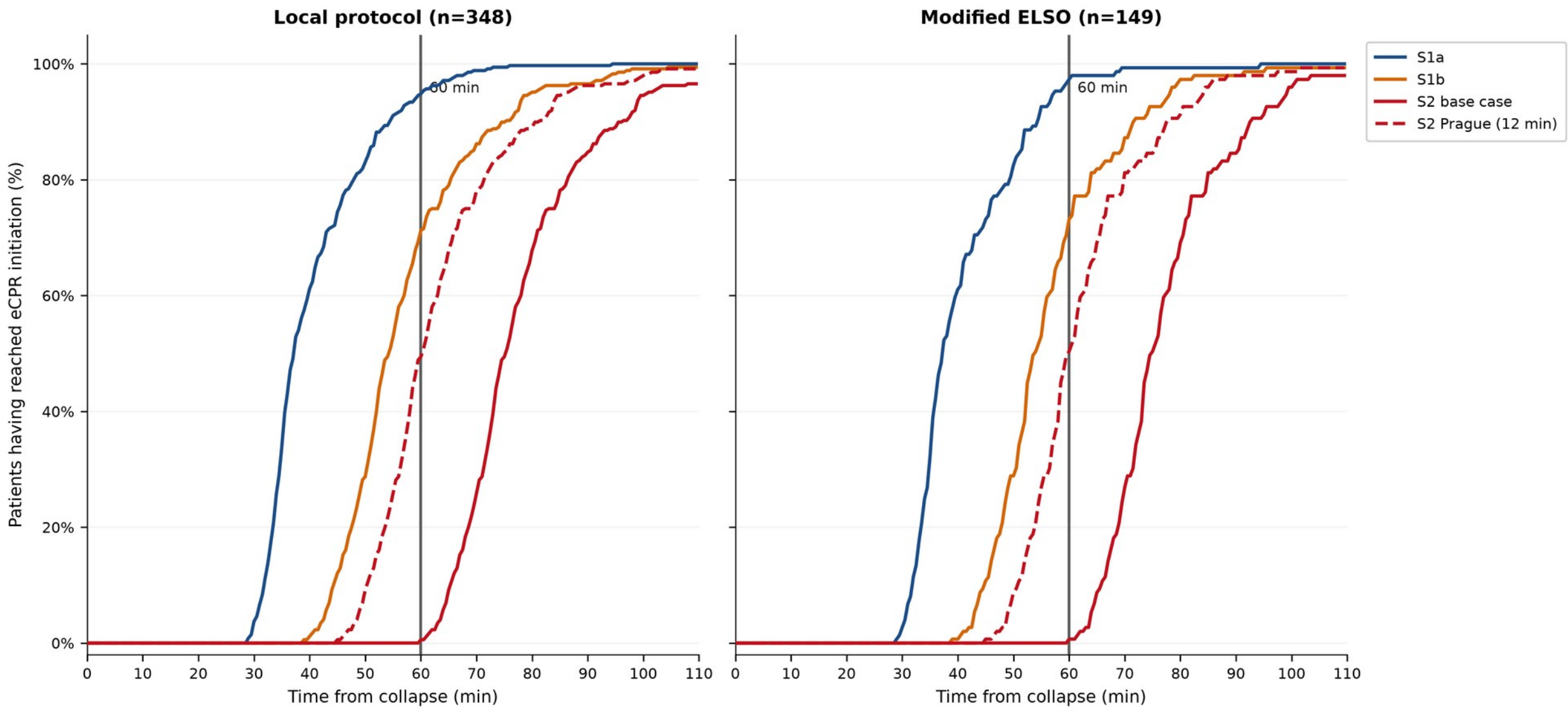

Supplementary Figure S5 *Sensitivity analysis in which the S2 scenario was recalculated using the in-hospital time-to-eCPR reported in the Prague OHCA study (median 12 min, IQR 11–14 min). This figure complements Supplementary Table S7.*

Supplementary Table S8 *ROSC-dependent endpoints under primary vs. alternative classification (local protocol cohort, n = 348)*

Endpoint	Primary: sustained ROSC only	Alternative: any documented ROSC
Eligible at hospital arrival, T = 10 min	188 (54.0%)	173 (49.7%)
Eligible at hospital arrival, T = 15 min	167 (48.0%)	149 (42.8%)
Eligible at hospital arrival, T = 20 min	150 (43.1%)	130 (37.4%)
S1a team dispatch at emergency call: still requiring eCPR AND ≤60 min	165 (47.4%)	143 (41.1%)
S1b team dispatch after eligibility assessment: still requiring eCPR AND ≤60 min	110 (31.6%)	96 (27.6%)
S2 in-hospital: still requiring eCPR AND ≤60 min	1 (0.3%)	0 (0.0%)

Primary classification: only sustained ROSC (admitted with ROSC) excludes a patient from requiring eCPR, using the documented ROSC time. Alternative classification: any documented ROSC at or before the decision point/hypothetical eCPR initiation excludes the patient at that moment, irrespective of later re-arrest. Patients with ROSC but undocumented timing are counted as still requiring eCPR in both classifications. Modified ELSO cohort behaves analogously (not shown).

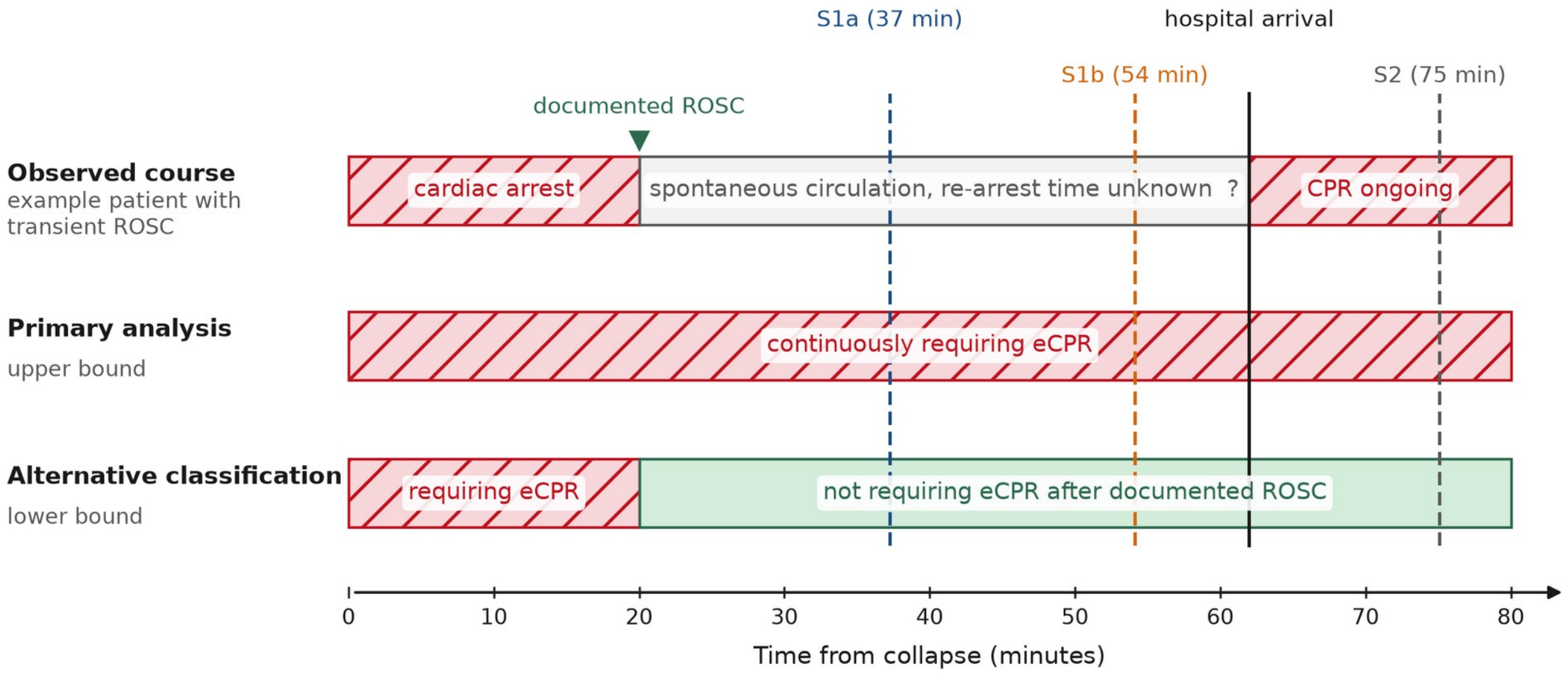

Supplementary Figure S6 *Handling of transient ROSC cases. Top: observed course of an example patient with transient ROSC — documented ROSC during prehospital care, re-arrest at an undocumented time, hospital arrival under ongoing CPR. As re-arrest times are not captured in the registry, the circulatory state between documented ROSC and hospital arrival is unknown at every modelled decision point (dashed lines: median time to hypothetical eCPR initiation, local protocol cohort; solid line: hospital arrival, the decision point of the primary analysis, at 62 min in this example). Middle: the primary analysis classifies such patients as continuously requiring eCPR; at every decision point they count as still requiring eCPR, yielding upper bounds — eligible at hospital arrival (T = 15 min): 48.0%; still requiring eCPR within 60 min: 47.4% (S1a), 31.6% (S1b), 0.3% (S2). Bottom: the alternative classification counts any documented ROSC at or before the decision point as not requiring eCPR at that moment, yielding lower bounds — 42.8% (arrival, T = 15 min); 41.1% (S1a), 27.6% (S1b), 0.0% (S2). All percentages refer to the local protocol cohort (n = 348; see Table above). The true proportions lie between the two bounds; conclusions are unchanged across this range.*

Supplementary Table S9 *Software and package versions used for all analyses*

Software / package	***Version***
Python	*3.10.16*
Quarto	*1.6.39*
numpy	*1.26.4*
pandas	*2.1.4*
scipy	*1.15.3*
statsmodels	*0.14.4*
matplotlib	*3.7.5*
tableone	*0.9.1*
geopandas	*1.0.1*
shapely	*2.0.7*

Bibliography

[1] Erblich R, Noitz M, Trimmel H, Voelckel W, Dos Reis Miranda D, Jenny D, et al. Eligibility and hypothetical time windows for eCPR in out-of-hospital cardiac arrest patients managed by a nationwide helicopter-emergency medical service: A multicentric, retrospective cohort study. Resusc Plus 2025;26:101160. https://doi.org/10.1016/j.resplu.2025.101160.

[2] Vos IA, Deuring E, Kwant M, Bens BWJ, Dercksen B, Postma R, et al. What is the potential benefit of pre-hospital extracorporeal cardiopulmonary resuscitation for patients with an out-of-hospital cardiac arrest? A predictive modelling study. Resuscitation 2023;189:109825. https://doi.org/10.1016/j.resuscitation.2023.109825.

[3] Grunau B, Reynolds J, Scheuermeyer F, Stenstom R, Stub D, Pennington S, et al. Relationship between Time-to-ROSC and Survival in Out-of-hospital Cardiac Arrest ECPR Candidates: When is the Best Time to Consider Transport to Hospital? Prehosp Emerg Care 2016;20:615–22. https://doi.org/10.3109/10903127.2016.1149652.

[4] Belohlavek J, Smalcova J, Rob D, Franek O, Smid O, Pokorna M, et al. Effect of Intra-arrest Transport, Extracorporeal Cardiopulmonary Resuscitation, and Immediate Invasive Assessment and Treatment on Functional Neurologic Outcome in Refractory Out-of-Hospital Cardiac Arrest: A Randomized Clinical Trial. JAMA 2022;327:737–47. https://doi.org/10.1001/jama.2022.1025
