# Supplementary material for: Modelling eCPR eligibility after out-of-hospital cardiac arrest: a 10-year retrospective temporospatial analysis from a mixed urban-rural region in Germany
Source: Scand J Trauma Resusc Emerg Med. 2026 Sep 9;34:159. doi: 10.1186/s13049-026-01681-7 (PMC13628803; doi:10.1186/s13049-026-01681-7)
Supplement: Supplementary file 2 [file 13049_2026_1681_MOESM2_ESM.pdf]

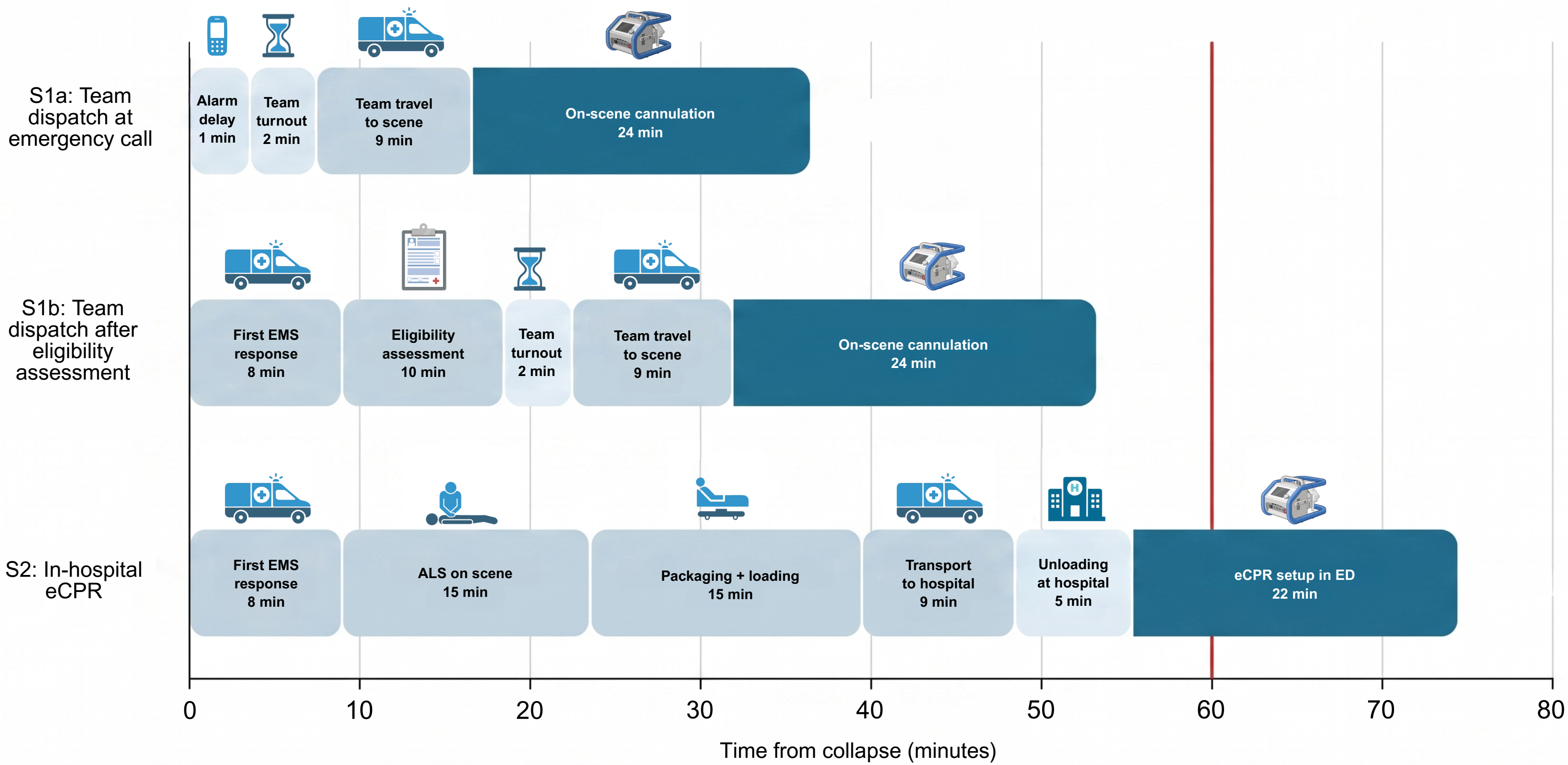
S1a: Team dispatch at emergency call
Alarm delay 1 min
Team turnout 2 min
Team travel to scene 9 min
On-scene cannulation 24 min
S1b: Team dispatch after eligibility assessment
First EMS response 8 min
Eligibility assessment 10 min
Team turnout 2 min
Team travel to scene 9 min
On-scene cannulation 24 min
S2: In-hospital eCPR
First EMS response 8 min
ALS on scene 15 min
Packaging + loading 15 min
Transport to hospital 9 min
Unloading at hospital 5 min
eCPR setup in ED 22 min
0
10
20
30
40
50
60
70
80
Time from collapse (minutes)
