# Supplementary material for: Modelling eCPR eligibility after out-of-hospital cardiac arrest: a 10-year retrospective temporospatial analysis from a mixed urban-rural region in Germany
Source: Scand J Trauma Resusc Emerg Med. 2026 Sep 9;34:159. doi: 10.1186/s13049-026-01681-7 (PMC13628803; doi:10.1186/s13049-026-01681-7)
Supplement: Supplementary file 3 [file 13049_2026_1681_MOESM3_ESM.pdf]

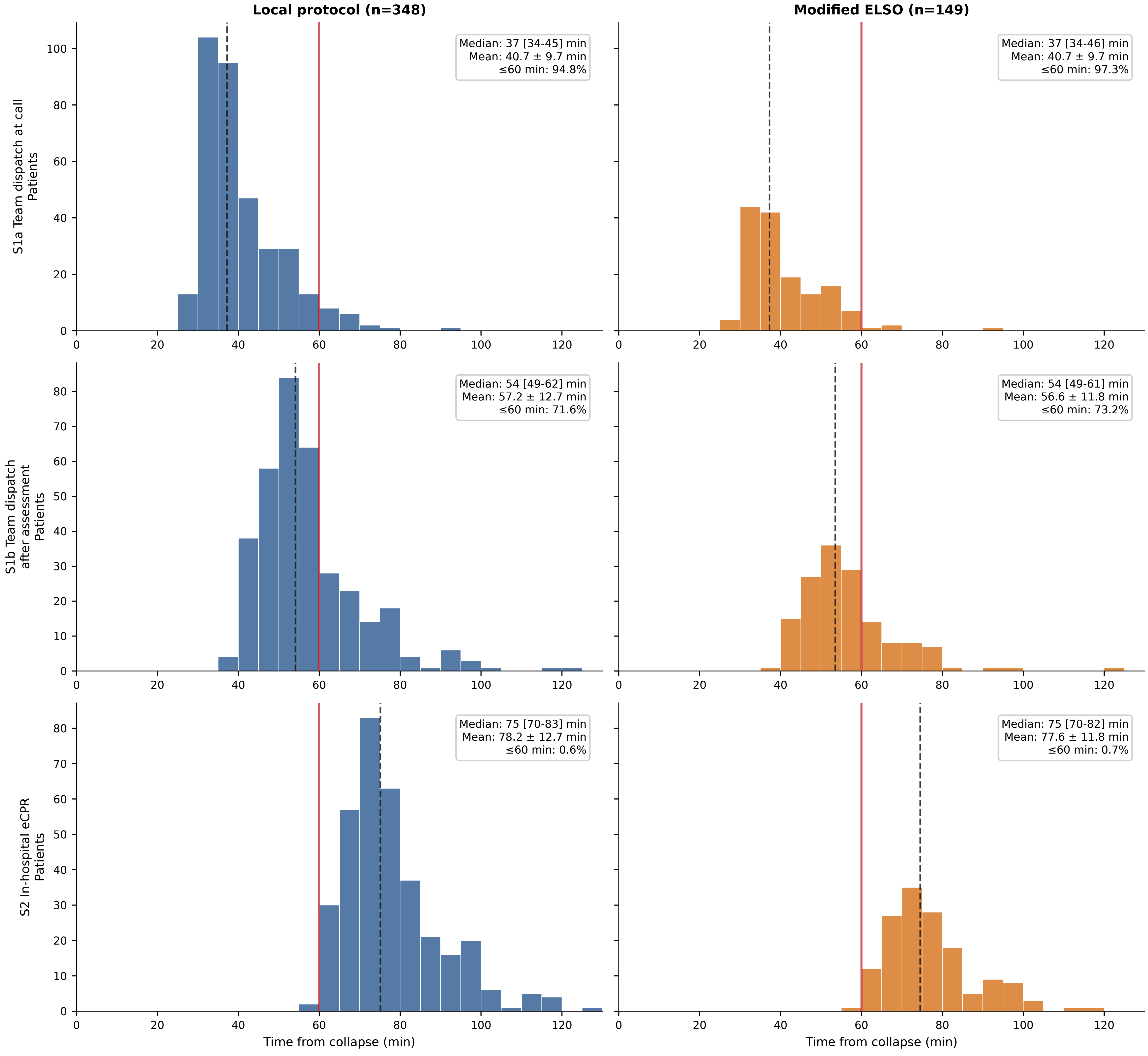
Local protocol (n=348)
Modified ELSO (n=149)
S1a Team dispatch at call
Patients
Median: 37 [34-45] min
Mean: 40.7 ± 9.7 min
≤60 min: 94.8%
Median: 37 [34-46] min
Mean: 40.7 ± 9.7 min
≤60 min: 97.3%
S1b Team dispatch
after assessment
Patients
Median: 54 [49-62] min
Mean: 57.2 ± 12.7 min
≤60 min: 71.6%
Median: 54 [49-61] min
Mean: 56.6 ± 11.8 min
≤60 min: 73.2%
S2 In-hospital eCPR
Patients
Median: 75 [70-83] min
Mean: 78.2 ± 12.7 min
≤60 min: 0.6%
Median: 75 [70-82] min
Mean: 77.6 ± 11.8 min
≤60 min: 0.7%
Time from collapse (min)
Time from collapse (min)
