# Supplementary material for: Modelling eCPR eligibility after out-of-hospital cardiac arrest: a 10-year retrospective temporospatial analysis from a mixed urban-rural region in Germany
Source: Scand J Trauma Resusc Emerg Med. 2026 Sep 9;34:159. doi: 10.1186/s13049-026-01681-7 (PMC13628803; doi:10.1186/s13049-026-01681-7)
Supplement: Supplementary file 5 [file 13049_2026_1681_MOESM5_ESM.pdf]

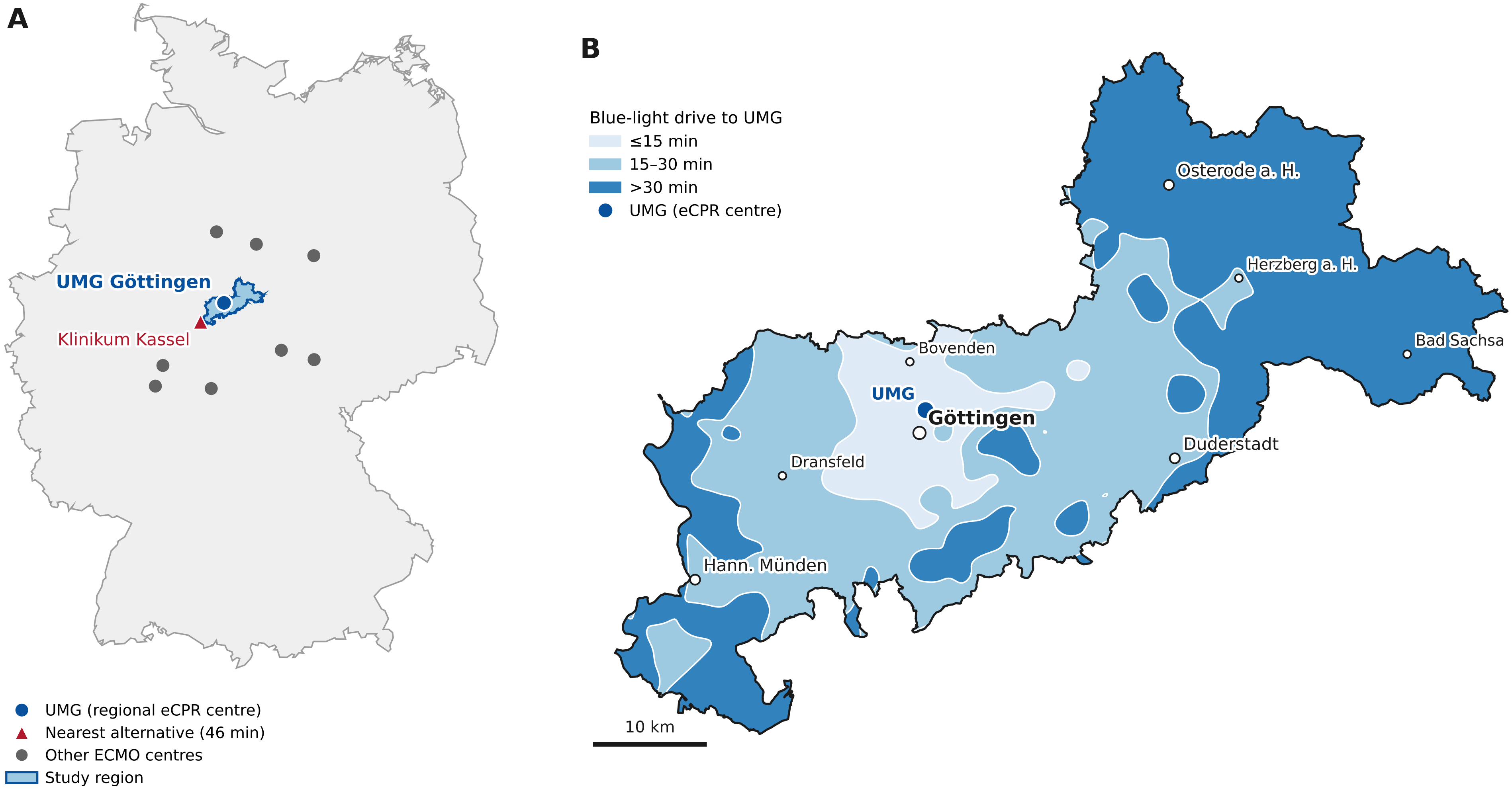

A
UMG Göttingen
Klinikum Kassel
UMG (regional eCPR centre)
Nearest alternative (46 min)
Other ECMO centres
Study region
B
Blue-light drive to UMG
≤15 min
15–30 min
>30 min
UMG (eCPR centre)
Osterode a. H.
Herzberg a. H.
Bad Sachsa
Bovenden
UMG
Göttingen
Duderstadt
Dransfeld
Hann. Münden
10 km
