# Supplementary material for: Modelling eCPR eligibility after out-of-hospital cardiac arrest: a 10-year retrospective temporospatial analysis from a mixed urban-rural region in Germany
Source: Scand J Trauma Resusc Emerg Med. 2026 Sep 9;34:159. doi: 10.1186/s13049-026-01681-7 (PMC13628803; doi:10.1186/s13049-026-01681-7)
Supplement: Supplementary file 6 [file 13049_2026_1681_MOESM6_ESM.pdf]

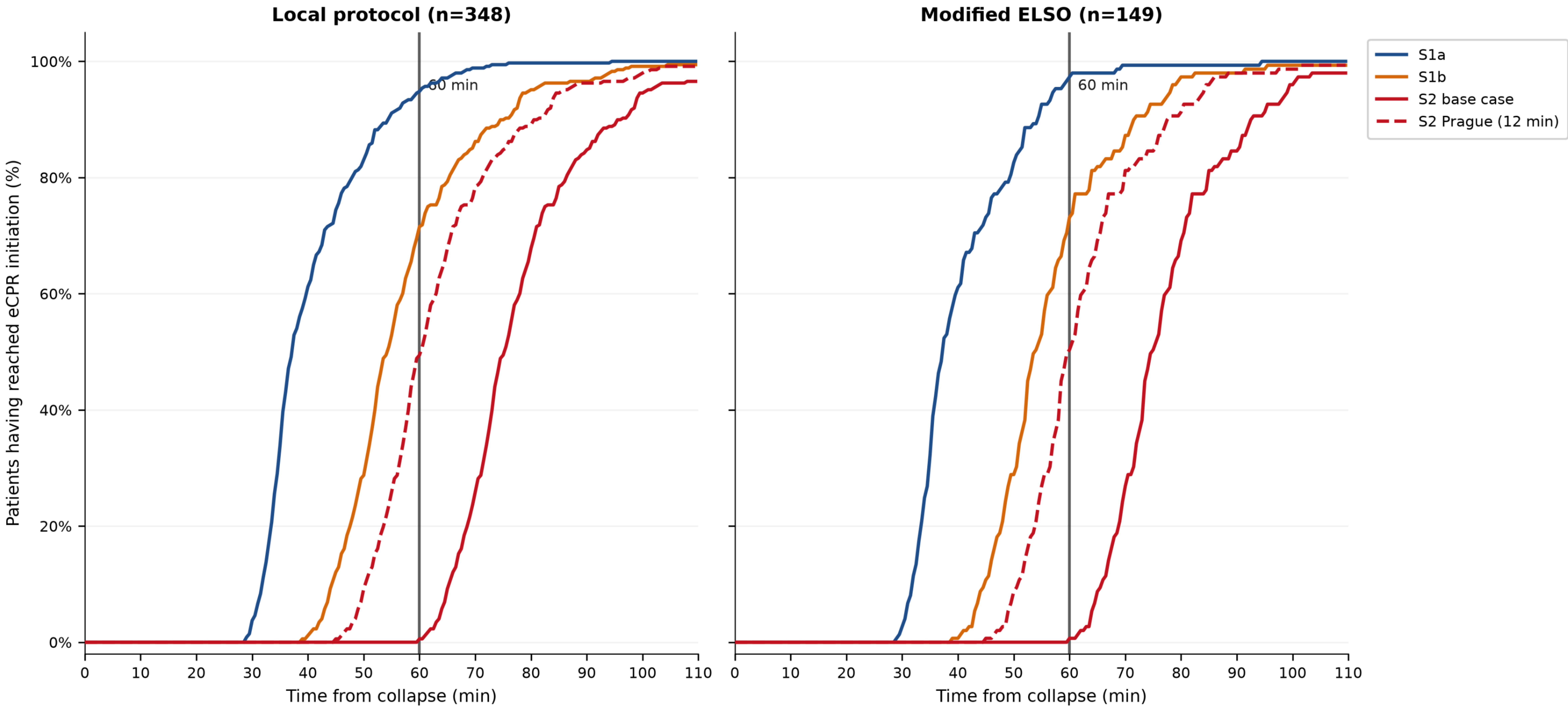

Local protocol (n=348)
Modified ELSO (n=149)
Patients having reached eCPR initiation (%)
100%
80%
60%
40%
20%
0%
60 min
60 min
0
10
20
30
40
50
60
70
80
90
100
110
Time from collapse (min)
Time from collapse (min)
S1a
S1b
S2 base case
S2 Prague (12 min)
