# Supplementary material for: Modelling eCPR eligibility after out-of-hospital cardiac arrest: a 10-year retrospective temporospatial analysis from a mixed urban-rural region in Germany
Source: Scand J Trauma Resusc Emerg Med. 2026 Sep 9;34:159. doi: 10.1186/s13049-026-01681-7 (PMC13628803; doi:10.1186/s13049-026-01681-7)
Supplement: Supplementary file 7 [file 13049_2026_1681_MOESM7_ESM.pdf]

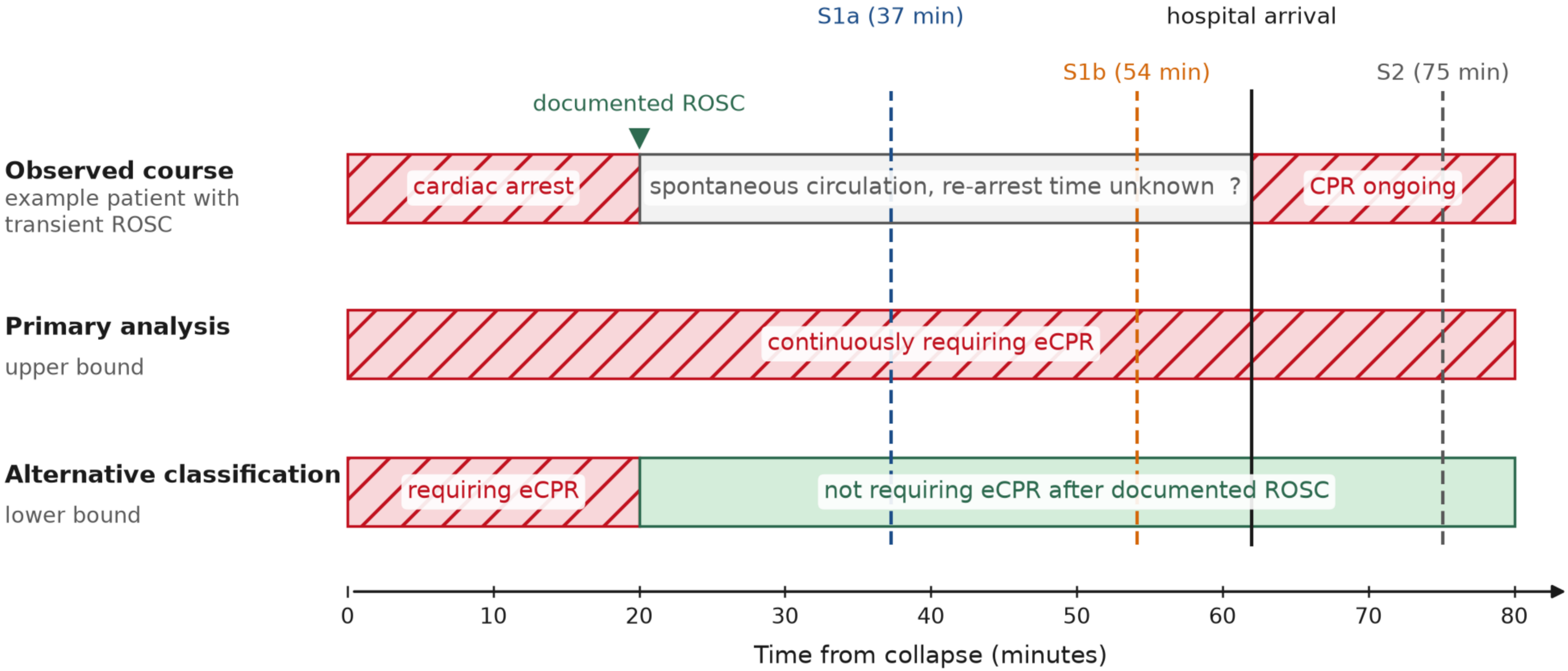
S1a (37 min)
hospital arrival
S1b (54 min)
S2 (75 min)
documented ROSC
Observed course
example patient with transient ROSC
cardiac arrest
spontaneous circulation, re-arrest time unknown ?
CPR ongoing
Primary analysis
upper bound
continuously requiring eCPR
Alternative classification
lower bound
requiring eCPR
not requiring eCPR after documented ROSC
0
10
20
30
40
50
60
70
80
Time from collapse (minutes)
